# E2F1/CDK5/DRP1 axis mediates microglial mitochondrial division and autophagy in the pathogenesis of cerebral ischemia‐reperfusion injury

**DOI:** 10.1002/ctm2.70197

**Published:** 2025-02-19

**Authors:** Ya‐Jing Yuan, Tingting Chen, Yan‐Ling Yang, Hao‐Nan Han, Li‐Ming Xu

**Affiliations:** ^1^ Department of Anesthesia Tianjin Medical University Cancer Institute &Hospital National Clinical Research Center for Cancer Tianjin's Clinical Research Center for Cancer Key Laboratory of Cancer Prevention and Therapy, Tianjin Tianjin China; ^2^ Department of Radiation Oncology Tianjin Medical University Cancer Institute &Hospital National Clinical Research Center for Cancer Tianjin's Clinical Research Center for Cancer Key Laboratory of Cancer Prevention and Therapy, Tianjin Tianjin China; ^3^ Hubei Key Laboratory of Tumor Microenvironment and Immunotherapy College of Basic Medical Sciences China Three Gorges University Yichang China

**Keywords:** CDK5, cerebral ischemia‐reperfusion, DRP1, E2F1, microglia, mitochondrial division, mitophagy, single‐cell transcriptome sequencing

## Abstract

**Background:**

The integrity of brain function is at stake due to cerebral ischemia‐reperfusion injury (CIRI), which encompasses mitochondrial dysfunction, autophagy, and neuroinflammation. The role of E2F1 in mediating these processes in microglia during CIRI remains unclear.

**Methods:**

A CIRI mouse model was utilized for single‐cell RNA transcriptome sequencing of brain tissues. The research comprised diverse gene expression, gene ontology (GO), and the enrichment of Kyoto Encyclopedia of Genes and Genomes (KEGG) pathways. Experimental techniques included oxygen‐glucose deprivation (OGD/R) cell models, RT‐qPCR, Western Blot, ChIP assays, and microglia‐neuron co‐cultures.

**Results:**

A significant aspect highlighted in the study was the involvement of CDK5 in the induction of mitochondrial abnormalities associated with CIRI. Upregulation of E2F1 and CDK5 in post‐CIRI microglia was observed. E2F1 facilitated CDK5 transcription, leading to DRP1 phosphorylation, exacerbating neurotoxic effects. Silencing E2F1 improved neurobehavioral outcomes in CIRI mice.

**Conclusions:**

Activation of E2F1‐mediated CDK5 drives mitochondrial division while inhibiting mitophagy in microglia, triggering inflammation, neuronal apoptosis, and exacerbating CIRI damage. Targeting this pathway could offer novel therapeutic strategies for mitigating CIRI‐induced brain injury.

**Key points:**

- Identification of the E2F1/CDK5/DRP1 Axis in CIRI This study reveals that the E2F1 transcription factor upregulates CDK5 expression, which in turn phosphorylates DRP1, promoting excessive mitochondrial fission and inhibiting mitophagy in microglia. This mechanism plays a critical role in cerebral ischemia‐reperfusion injury (CIRI).
- Mitochondrial Dysfunction and Neuroinflammation The activation of DRP1 leads to mitochondrial fragmentation and excessive ROS accumulation, triggering microglial activation and inflammatory responses, exacerbating neuronal apoptosis and brain injury in CIRI.
- Therapeutic Potential of E2F1 Silencing Knockdown of E2F1 in microglia effectively reduces mitochondrial damage, restores mitophagy, suppresses inflammation, and improves neurological outcomes in a CIRI mouse model, highlighting a promising therapeutic target for ischemic stroke intervention.

## Graphical abstract

1. E2F1‐mediated CDK5 activation drives mitochondrial dysfunction in microglia, exacerbating CIRI damage.
2. Mitochondrial fission and impaired mitophagy are key processes influenced by E2F1‐CDK5 signalling in CIRI.
3. Targeting the E2F1‐CDK5‐DRP1 axis offers novel therapeutic strategies for mitigating CIRI‐induced brain injury.

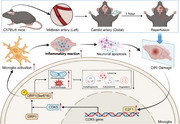

## 1 INTRODUCTION

The phenomenon of cerebral ischemia‐reperfusion injury (CIRI) is a widespread issue within neurology and is associated with conditions encompassing coronary heart disease and cerebrovascular disease, serving as a significant cause of brain function impairment and mortality.^1^, ^2^ Ischemic stroke stands out as a prominent disease on a global scale, known for its elevated levels of fatality and impairment.^3^ Patients suffering from ischemic stroke might encounter CIRI subsequent to the reestablishment of blood circulation, worsening neuronal fatality, and neurological impairment, consequently hindering the rehabilitation process of individuals with acute ischemic stroke.^4^ The interruption of cerebral blood flow followed by reperfusion leads to severe cellular damage due to hypoxia and oxidative stress during the reperfusion process, triggering a cascade of pathophysiological changes such as apoptosis, inflammatory responses, and other processes.^5^, ^6^, ^7^ The evolution of CIRI is intertwined with diverse immune cells that have a dual function in driving its advancement. Post‐CIRI, immune cells like microglia and macrophages produce tissue‐mending elements and engulf expired cells to assist in brain tissue regeneration.^8^ Despite the identification of certain molecular pathways linked to CIRI in prior research, the pathophysiological mechanisms of this condition are intricate and not fully grasped.^9^, ^10^, ^11^ Currently, there are no efficient medications accessible to manage or avoid CIRI.^12^ Consequently, additional investigation is required to pinpoint potential medications for managing CIRI.

Mitochondria, as the energy production centres and crucial cell regulators within the cellular environment, play vital roles in CIRI.^5^, ^13^, ^14^ Brain tissues have high demands for oxygen and nutrients, with mitochondria being the essential energy source to maintain normal cellular functions.^15^ However, during cerebral ischemia‐reperfusion, interrupted blood flow leads to insufficient oxygen and nutrient supply to cells, resulting in mitochondrial dysfunction.^16^ This anomaly manifests as a reduction in ATP synthesis, leading to insufficient cellular energy supply, consequently affecting the normal physiological functions of the cell.^17^ Additionally, the escalation of oxidative stress reactions in mitochondria results in a boost in the generation of reactive oxygen species (ROS), triggering intracellular oxidative stress and causing harm to biomolecules including cell membranes, proteins, and nucleic acids.^18^, ^19^ Furthermore, cerebral ischemia‐reperfusion also results in the loss of mitochondrial membrane potential, disrupting the normal structure and function of mitochondria.^20^, ^21^ These mitochondrial dysfunctions lead to cell death and exacerbate tissue damage, worsening the extent of CIRI.^22^, ^23^, ^24^ Therefore, profound knowledge of the distinct functions and governing systems of mitochondria in CIRI is essential in averting and managing this issue.^25^, ^26^ By elucidating the role of mitochondria in CIRI, we can better understand this disease's pathogenesis, providing new targets and strategies for clinical therapy.^27^, ^28^

The crucial participation of the transcription factors E2F1 and CDK5 in the nervous system, notably in CIRI, has been highlighted by recent investigations.^29^ E2F1, a significant cell cycle regulatory factor, is involved in cell proliferation and cycle regulation and influences diverse biological functions including cell apoptosis, DNA damage repair, and inflammatory responses. In the nervous system, the expression of E2F1 is closely associated with neuronal development, synapse formation, and neuronal apoptosis.^30^, ^31^ Additionally, E2F1 has been found to play crucial roles in various neurological disorders, encompassing stroke, Parkinson's disease, and Alzheimer's disease.^32^, ^33^ CDK5, a specific neuronal kinase, is crucial for the growth and plasticity of neuronal cells.^34^, ^35^, ^36^ By phosphorylating various substrate proteins, CDK5 participates in regulating processes like neuronal movement, synaptic transmission, and neuron migration.^37^, ^38^ However, the exact mechanisms of action of E2F1 and CDK5 in CIRI remain incompletely understood.^39^, ^40^ Investigations have hinted at the potential involvement of E2F1 and CDK5 in the initiation and advancement of CIRI by modulating signalling pathways related to mitochondrial function, cell apoptosis, and inflammatory responses. Therefore, a thorough investigation into the mechanisms of action of E2F1 and CDK5 in CIRI is crucial for understanding the pathogenesis of this disease and identifying fresh avenues for therapeutic intervention.

Investigating the fundamental aim is to delve into the molecular pathway by which E2F1 regulates CDK5‐DRP1 phosphorylation to mediate microglial mitochondrial division and autophagy, thereby impacting CIRI. By combining mouse models, in vitro cell experiments, and techniques such as Single‐cell transcriptome sequencing, we will systematically analyze the roles of E2F1 and CDK5 in CIRI and their molecular regulatory mechanisms. The primary goal of this investigation is to advance insight into the pathogenesis of CIRI, offering new targets and strategies for treating related diseases. Ultimately, our goal is to provide scientific evidence for the prevention and treatment of CIRI by elucidating the mechanisms of action of these key molecules and pathways, thus improving patients’ quality of life with significant clinical importance.

The conclusions drawn from this research will enhance comprehension regarding the pathogenesis of CIRI, providing new insights and methods for treating the condition. By clarifying the roles of E2F1 and CDK5 in CIRI, we hope to identify new drug targets and formulate enhanced treatment regimens to ease patients' agony and boost their life quality. Therefore, this research carries substantial scientific and clinical relevance and potential, offering new perspectives and methods to address the serious health issue of CIRI.

## 2 METHODS

### 2.1 Research subjects

Sourced from Jackson Laboratory, C57BL/6J male mice (aged 8 weeks, weighing 22–25 g) and Cx3cr‐Cre mice (C57BL/6J background) were obtained with stock numbers 000664 and 025524. Lodged in a specific pathogen‐free (SPF) animal centre with regulated humidity (60–65%) and temperature (22–25°C), the mice were accommodated. They were provided with standard lab chow (5–10 g/day) and ad libitum access to water (8–15 mL/day). Upon completion of a 7‐day acclimatization phase, the experiments commenced following an assessment of the mice's health status. The institution's Animal Ethics Committee granted approval for the experimental processes and animal treatment to minimize potential adverse effects on the animals, ensuring the scientific merit and necessity of the study. The ethical principles and relevant regulations governing animal experimentation were strictly adhered to in order to safeguard the well‐being and rights of the animals.

### 2.2 Establishment of the CIRI mouse model

Experimental induction of transient focal brain ischemia in mice was achieved by temporarily occluding the left middle cerebral artery through the intraluminal suture method. Specifically, the anaesthesia of C57BL/6 mice involved the application of 1.2% isoflurane, with careful maintenance of body temperature at 37°C while inducing ischemia. An incision along the midline of the neck provided visibility of the left external carotid artery, internal carotid artery, and common carotid artery during the surgical procedure. Subsequently, surgical procedure executed at the terminal segment of the common carotid artery, succeeded by the introduction of a nylon suture with a diameter range of .25 ± .03 mm to a specific depth. Laser Doppler flowmetry (LDF, FLPI2, Moor) was employed for the real‐time monitoring of cerebral blood flow, and reperfusion was performed after 1 h ischemia. The identical surgical procedure was carried out on mice in the sham group without inducing vessel occlusion.

### 2.3 Transcriptome sequencing of brain tissues

Transcriptome profiling of brain tissues from six CIRI mice and six Sham group mice was conducted using high‐throughput RNA sequencing. Initially, total RNA isolation was performed utilizing Trizol reagent (Invitrogen) followed by quantification and assessment of RNA purity through a Nanodrop 2000 spectrophotometer (Thermo Fisher Scientific). Carefully selected RNA specimens conforming to the outlined guidelines were earmarked for further exploration: RNA Integrity Number (RIN) ≥7.0 and 28S:18S ratio ≥1.5.

The libraries were produced and sequenced under the supervision of CapitalBio Technology situated in Beijing, China. The RNA quantity for each sample was 5 micrograms. The Ribo‐Zero Magnetic Kit (Epicentre Technologies) depletes ribosomal RNA (rRNA) from the total RNA. NEB Next Ultra RNA Library Prep Kit (NEB) was applied in the generation of Illumina libraries intended for sequencing purposes. Later, the RNA fragments were specifically picked to achieve a size of around 300 bp in the NEB next first strand synthesis reaction buffer (5×). The initial formation of the primary cDNA strand involved the utilization of reverse transcriptase priming sequences and random starting primers, whereas the generation of the secondary cDNA strand occurred within a reaction medium containing dUTP mix (10×). Processing of the cDNA fragments involved end repair actions, such as appending polyA tails and connecting sequencing adapters by ligation. After the attachment of Illumina sequencing adapters, the USER enzyme (NEB) was deployed to degrade the secondary cDNA strand in order to produce libraries that are specific to individual strands. Library DNA underwent amplification, purification, and PCR‐based enrichment, followed by identification using the Agilent 2100 platform, and the quantification process was executed with the KAPA Library Quantification Kit (KAPA Biosystems). Eventually, the NextSeq 500 sequencer (from Illumina) was employed for executing paired‐end sequencing.

### 2.4 Ensuring the accuracy of sequencing data and mapping to the reference genome

Examination of the excellence of the paired‐end reads within the initial sequencing data was undertaken with FastQC software version 0.11.8. Processing of the raw dataset involved the utilization of Cutadapt version 1.18, which aimed to eliminate Illumina sequencing adapters and poly(A) tail sequences. Implementation of a Perl script resulted in the exclusion of readings surpassing 5% N content. The extraction process, driven by FASTX Toolkit software 0.0.13, targeted reads meeting a minimum base quality of 20, making up 70%. BBMap software aided in rectifying the paired‐end sequence inaccuracies. Following this, the screened superior read segments were mapped to the reference genome with the aid of HISAT2 software (0.7.12).

### 2.5 Transcriptome sequencing data analysis

Significantly differentially expressed genes (DEGs) were selected based on the transcriptome sequencing data through the use of the “limma” package in R, employing a threshold criterion of *p*‐value < .05 and |log2FC| > 1. Subsequently, within the R programming framework, enrichment analysis for the DEGs incorporating gene ontology (GO) and Kyoto Encyclopedia of Genes and Genomes (KEGG) was accomplished through the “ClusterProfiler” module, while the visualization of the obtained enrichment outcomes was achieved via the utilization of the “ggplot2” tool. E2F1 target gene binding sites were predicted on the hTFtarget website. The top 25 genes showing the most significant upregulation and downregulation were individually imported into the String database (https://string‐db.org) for examining protein–protein interactions (PPI), leading to the export of the PPI protein node file. Using the R software, the construction of a statistical graph relied on the node data extracted from the PPI network. Finally, the core gene, characterized by its extensive interactions with multiple nodes in the network, was singled out for detailed analysis.

### 2.6 Single‐cell transcriptome sequencing and data examination

The brain tissues from CIRI mice (N1 and N2) and Sham group mice (N3 and N4) were washed in cold PBS, followed by digestion with 1 mg/mL collagenase (C2674, Sigma‐Aldrich) lasting 10 min at 37°C. Afterwards, a single‐cell mixture was produced by treating the samples with trypsin/EDTA (25200072, Gibco) at a temperature of 37°C for a duration of 5 min. Isolation of single cells was accomplished through the C1 Single‐Cell Auto Prep System provided by Fluidigm, Inc. After cell trapping, mRNA was released by cell lysis on the chip, succeeded by cDNA synthesis applying the reverse transcription technique. The pre‐amplification of cDNA post‐lysis and reverse transcription was done on a microfluidic chip to enable sequencing. The produced cDNA was utilized for the formation of libraries designed for single‐cell sequencing on the HiSeq 4000 Illumina sequencing platform.

Utilization of the Seurat software suite facilitated the examination of the scRNA‐seq data. The initial step involved implementing measures to ensure data quality, with specific conditions set: nFeature_RNA > 500 & nCount_RNA > 3000 & nCount_RNA < 20000 & percent.mt < 5. Post LogNormalize standardization, the RunPCA function executed principal component analysis (PCA) on the leading 2000 genes displaying notable diversity. Key PCs were picked for UMAP clustering analysis using JackStrawPlot and ElbowPlot functions. Cell clusters were distinguished by identifying markers through the application of the FindAllMarkers function, followed by cell annotation utilizing the CellMarker database. Violin plots were generated using the VlnPlot function to demonstrate the variation in marker gene expression among diverse cell clusters.

### 2.7 Lentivirus construction

Lentivirus‐based gene interference and overexpression vectors targeting E2F1 and CDK5 genes were developed through acquiring lentiviral interference vector pSIH1‐H1‐copGFP (sh‐, SI501A‐1, System Biosciences) and lentiviral overexpression vector pCDH‐CMV‐MCS‐EF1α‐copGFP (oe‐, CD511B‐1, System Biosciences). The lentivirus packaging kit (A35684CN, Invitrogen) facilitated the transduction of HEK‐293T cells with lentiviral vectors containing the prescribed sequences provided by Saibai Kang Biological Technology Co., Ltd. (catalogue number: iCell‐h237). The lentivirus was detected in the cell culture supernatant obtained 48 h after packaging, showing a titer of 1 × 10^8^ TU/ml. The following sequences were utilized: sh‐NC sequence: TACGAACGAGACCTCACTGAATT; sh‐E2F1‐1 sequence: GCCAAGAAGTCCAAGAATCAT; sh‐E2F1‐2 sequence: GATCTCTTTGACTGTGACTTT. The sequence for sh‐CDK5 was GGAGATCTGTCTACTCAAA (RiboBio).

### 2.8 Establishment of OGD/R cell model

The oxygen‐glucose deprivation (OGD) method was used to simulate ischemic conditions in vitro for microglial cells. In brief, BV2 mouse microglial cells (catalogue no.: MZ‐0280, Ningbo Mingzhou Biotechnology Co., Ltd.) were cultured in serum‐ and glucose‐free DMEM (catalogue no.: 11966025, ThermoFisher) and subjected to 2 h of hypoxic incubation in a sealed hypoxia chamber, after which they were cultured at 37°C for 24 h in DMEM/F‐12 (catalogue no.: 11320082, ThermoFisher) supplemented with 10% FBS (catalogue no.: 10099158, ThermoFisher).

### 2.9 Cell grouping and treatment

Different groups were established for BV2 cells, including oe‐NC, oe‐E2F1, sh‐NC, sh‐E2F1, sh‐NC+oe‐NC, sh‐E2F1+oe‐NC, and sh‐E2F1+oe‐CDK5. Each group of cells was inoculated with the corresponding lentivirus and cultured for 48 h to evaluate the infection efficiency. The experimental design included the following groups: Control, OGD/R, OGD/R+Roscovitine, OGD/R+Mdivi‐1, OGD/R+NAC (1 mmol/L), and OGD/R+NAC (10 mmol/L). The treatment involved using 15 µmol of the CDK5 inhibitor Roscovitine (product code: HY‐30237, MCE), 10 µmol of the dynamin‐related protein 1 (DRP1) inhibitor Mdivi‐1 (Product Code: HY‐15886, MCE), and the ROS scavenger NAC (product code: HY‐B0215, MCE) to treat the cells separately to establish the OGD/R cellular model. For the OGD/R+sh‐NC and OGD/R+sh‐CDK5 groups, a culture of the cells with the corresponding lentivirus was continued for 48 h, and then the infection efficiency was evaluated following the aforementioned method to construct the OGD/R cellular model. The DRP1‐S616A group utilized the mutant form DRP1‐S616A to enhance DRP1 activity.^13^, ^41^

### 2.10 RT‐qPCR

Utilization of TRIzol reagent (ThermoFisher) allowed for total RNA extraction from tissues and cells, with subsequent evaluation of RNA concentration and purity through the utilization of a NanoDrop 2000 spectrophotometer (ThermoFisher). Utilizing the PrimeScript RT reagent Kit (Takara; Code: RR047A), the mRNA was transcribed in reverse to produce cDNA in accordance with the prescribed procedures. TaKaRa designed gene‐specific primers for the study (Table S1). Utilizing the 7500 fast real‐time PCR system (ThermoFisher), real‐time quantitative PCR was executed following these steps: starting with an initial denaturation at 95°C for 10 min, denaturation at 95°C for 10 s, annealing at 60°C for 20 s, extension at 72°C for 34 s, repeated for 40 cycles. GAPDH functioned as a reference gene, and the assessment of the relative transcription level of the target gene involved the use of the comparative ΔΔCt methodology: ΔΔCt = ΔCt experimental group − ΔCt control group, where ΔCt = Ct (target gene) − Ct (reference gene), and the target gene mRNA's relative transcription level was calculated as 2^−ΔΔCt^. The experiment was replicated thrice.

### 2.11 Cellular immunofluorescence

Cultivation in immunofluorescence chambers involved seeding cells at a density of 2 × 10^5^ cells per well and continuing until achieving a fusion rate of around 90%. Upon reaching this threshold, the cells underwent triple rinsing with PBS, followed by fixation in 4% paraformaldehyde (1 mL per well) and incubation at room temperature (RT) lasting 15 min. Cell permeabilization was attained by exposure to .3% Triton X‐100 for 10 min after a series of three PBS washes, succeeded by three more PBS washes. Subsequently, after blocking with goat serum (catalogue number: 16210072, ThermoFisher) for 30 min and triple PBS rinses, the cells were exposed to a night‐long incubation at 4°C with primary antibodies against E2F1 (1:500, NBP2‐56716, novusbio) and CDK5 (1:100, NBP2‐15843, novusbio) in PBS. Following three additional rinses using PBS, a secondary antibody Goat anti‐Rabbit IgG H&L (Alexa Fluor 647; 1:200, ab150079, Abcam) was applied to the cells for 1 h at RT in the absence of light. Upon three PBS washes, the cells received DAPI staining in darkness for 15 min, followed by a triple rinse with PBS and sealing using a fluorescent mounting medium (catalogue number: P36961, ThermoFisher) before observation and imaging using a fluorescence microscope. For ROS detection, microglia cells were incubated with 5 µM CellROX Deep Red (catalogue number: C10443, ThermoFisher) for 30 min, underwent three PBS washes, DAPI staining lasting 5 min, observation and imaging with a fluorescence microscope, and finally, quantification of fluorescence intensity using the ImageJ image analysis software.

### 2.12 Tissue immunofluorescence

The brain tissue sections were processed for antigen retrieval with sodium citrate buffer after deparaffinization and rehydration, lasting 5 min. Afterwards, blocking of nonspecific antibody adherence was achieved through a 60 min incubation with 5% goat serum on the sections. Primary antibodies against E2F1 (NBP2‐56716, novusbio), CDK5 (1:200, NBP2‐15843, novusbio), DRP1 (1:500, NB110‐55288, novusbio), the microglial marker Iba‐1 (1:100, ab283346, Abcam), and the mitochondrial marker COX IV (1:100, ab210675, Abcam) were introduced for overnight incubation at 4°C. Subsequently, secondary antibodies Goat anti‐Rabbit IgG H&L (Alexa Fluor 647; 1:500, ab150079, Abcam), Goat anti‐Rabbit IgG H&L (Alexa Fluor 488; 1:200, ab150077, Abcam), and Goat anti‐Rat IgG H&L (Alexa Fluor 488; 1:200, ab150165, Abcam) were used for incubation. Following a 1 h incubation at RT without light exposure, samples underwent staining with the nuclear dye DAPI for 5 min in the absence of light, before assessing fluorescence intensity utilizing the Olympus BX51 fluorescence microscope (Olympus Corp), paired with the image processing software, ImageJ.

### 2.13 Dual‐luciferase assay

Based on the overexpression and knockdown of E2F1 lentivirus in 293T cells (oe‐NC, oe‐E2F1, sh‐NC, sh‐E2F1), they underwent transfection with a dual luciferase reporter gene vector incorporating the CDK5 promoter sequence (TTTGGCGG) and its mutant variant (AAACCGCC) at the binding sites through Lipofectamine 2000 reagent kit (catalogue no: 11668019, ThermoFisher). The focus was on examining how E2F1 affects the transcriptional activity of the CDK5 promoter. After the transfection process, cell samples were gathered and subjected to lysis within 2 days, then assessed utilizing a luciferase detection assay kit (K801‐200, Biovision). Employing the dual‐luciferase reporter gene analysis system (Promega) facilitated the examination of the luciferase reporter gene. The internal reference gene presence was attributed to Renilla luciferase and evaluation of the target reporter gene's activation level involved comparing the ratio of Firefly luciferase assay value (RLU) with Renilla luciferase assay value (RLU).

### 2.14 ChIP experiment

An evaluation of E2F1 enrichment in the CDK5 gene promoter site was conducted employing the ChIP assay kit (catalogue number: KT101‐02, Sai Cheng Biotechnology Co., Ltd.). The specific protocol involved culturing cells until they reached 70–80% confluency, followed by adding 1% formaldehyde for fixation at ambient conditions for a duration of 10 min to crosslink DNA and proteins within the cells. Postfixation, the cells underwent random shearing through sonication, with cycles of 10 s pulses at 10 s intervals repeated 15 times to fragment the DNA into suitable sizes. Subsequently, the lysate was subjected to centrifugal forces of 13 000 revolutions per minute under a temperature of 4°C, leading to the separation of the supernatant into two individual tubes. A negative control antibody, Rabbit IgG (ab172730, 1:100, Abcam) was introduced to one tube for incubation, with the specific antibody Rabbit anti‐E2F1 (1:100, NBP3‐15785, novusbio) being added to the other tube, overnight at RT. Utilizing protein agarose/sepharose led to the precipitation of DNA–protein complexes, and eliminating the supernatant quickly with centrifugation led to the removal of non‐specific complexes, followed by overnight de‐crosslinking at 65°C. After the extraction process involving phenol/chloroform, DNA fragments were purified to retrieve the DNA segments essential for analyzing the CDK5 gene promoter region through qPCR. The utilized primer sequences were forward: 5′‐ATCTAGGCCGTCAAACGCAA‐3′ and reverse: 5′‐GTGTGCCCCGCTCTTGTTAT‐3′.

### 2.15 Western blot

The isolation of total protein, membrane protein, and mitochondrial protein from tissues or cells was carried out utilizing RIPA lysis buffer encompassing PMSF (P0013C, Beyotime). Protein extraction involves centrifugation to separate organelles and membrane structures. Initially, homogenization of tissue or cell samples was performed, followed by treatment with RIPA lysis buffer and subsequent removal of cellular debris through low‐speed centrifugation. Harvested was the supernatant, where membrane proteins (found in the supernatant) and mitochondrial proteins (in the pellet) were separated by high‐speed centrifugation. Incubation of the extracted proteins on ice for 30 min at 4°C was conducted, followed by centrifugation at 8000*g* for 10 min to separate the supernatant. The BCA assay kit (ThermoFisher, USA; catalogue no: 23227) was employed for determining the total protein concentration. The sequential procedure comprised dissolving 50 µg of protein in 2× SDS loading buffer, applying heat to the mixture at 100°C for 5 min, executing SDS‐PAGE gel electrophoresis, and transferring the protein onto a PVDF membrane. Blocking of the PVDF film required the application of 5% skimmed milk for 1 h at RT, followed by detection using primary antibodies that were suitably diluted including p‐DRP1(Ser616) (1:1000, ab314756, Abcam), DRP1 (1:2000, ab156951, Abcam), CDK5 (1:1000, ab40773, Abcam), AOM20 (1:1000, Abcam), TIM23 (1:1000, 11123‐1‐AP RoteinTech), β‐actin (ab8226, 1:1000, Abcam), COX IV (ab202554, 1:2000, Abcam), Tubulin (ab7291, 1:5000, Abcam), and GAPDH (ab8245, 1:5000, Abcam). After an overnight stay at 4°C, the membrane went through three rounds of TBST washing, with each wash lasting 10 min. Subsequently, it was treated with an HRP‐conjugated secondary antibody, Goat Anti‐Rabbit IgG H&L (HRP; ab97051, diluted 1:2000, Abcam) for 1 h. After the TBST rinsing steps, the clean glass plate served as the base for the membrane positioning. A moderate quantity of Solutions A and B obtained from the ECL chemiluminescent detection kit (catalogue no: abs920, Aibit Biotech) were combined in low light conditions, applied onto the membrane, and recorded with the Bio‐Rad imaging system (Bio‐Rad). The analytical process involved the use of Quantity One v4.6.2 software, whereby the grayscale value of the protein bands was standardized to the internal control protein band for the determination of relative protein levels accurately. The experiment was executed in triplicate, and subsequent computation yielded the average values.

### 2.16 Evaluation of ROS levels in cells and mitochondria

Cellular and mitochondrial reactive oxygen species (Mito‐ROS) levels were assessed via the Dichlorodihydrofluorescein Diacetate (DCFH‐DA) staining method. DCFH‐DA (catalogue number: C2938, ThermoFisher, USA) was diluted to 10–20 µM prior to its introduction into the cells. Incubation of the cells was carried out at 37°C lasting between 20 and 30 min, allowing DCFH‐DA to enter the cells and be metabolized. Following incubation, PBS was utilized for washing to eliminate excess DCFH‐DA and fresh culture medium was added for further incubation at 37°C to enable the reaction of DCFH‐DA with ROS, resulting in green fluorescence. Finally, fluorescence intensity was examined using a fluorescence microscope.

Analysis of MitoSOX live‐cell imaging fluorescence outputs enabled the quantification of Mito‐ROS levels. The confocal microscopy (Leica TCS SP5, Wetzlar) captured the red fluorescence from MitoSOX within microglia under 580 nm excitation and 510 nm emission wavelengths. Assessment of the average red fluorescence intensity produced by MitoSOX was carried out via the ImageJ application.

### 2.17 Measurement of autophagic flux

Ad‐mRFP‐GFP‐LC3 (Beyotime Biotechnology) transfection was performed on microglia over a 24 h period to evaluate autophagic flux. Intense red fluorescence of LC3 protein indicates autophagosomes have been engulfed in the lysosomal acidic environment, forming autolysosomes, indicating unimpeded mitophagy flux; strong green fluorescence of LC3 protein suggests autophagosomes have not converted to autolysosomes, implying autophagy is blocked. The quantities of red and green punctate structures were calculated using ImageJ's image analysis software.

### 2.18 Cerebral blood flow spot CT scan

Following anaesthesia, Sham and CIRI mice were placed prone on a board to fully expose the scalp. The positioning of a low‐energy helium‐neon laser probe 14 cm above the skull of the mouse allowed for the visualization of blood flow by applying a laser Doppler perfusion imager (PeriCam PSI ZR). Subsequently, blood flow measurements were conducted employing the blood flow analysis software PIMsoft (version 1.5, Perimed AB).

### 2.19 Cellular and brain tissue mitochondrial morphology

The labelling of microglia's mitochondria was accomplished by utilizing MitoTracker Deep Red (100 nmol/L) for a duration of 30 min at 37°C, followed by observation through confocal microscopy (Leica TCS SP5, Wetzlar). Stimulating mitochondrial fluorescence clusters involved a 633 nm laser, with the detection of fluorescence emitted between 558 and 617 nm. The measurement of mitochondrial length was analyzed and computed utilizing the ImageJ software. Fresh brain tissue samples were processed and inspected by a transmission electron microscope (TEM; H‐7500, Hitachi). The brain tissue's mitochondrial length and cristae density were quantified using ImageJ software.

### 2.20 ELISA

The cellular culture media from each group were subjected to ELISA testing adhering to the guidelines set forth by the producer to quantify the concentrations of inflammatory markers. Utilized in the experiment were the ELISA kits as listed below: IL‐1β (catalogue number: JL18442‐48T, Future Industries Co., Ltd.), IL‐6 (catalogue number: JL20268‐48T), TNF‐α (catalogue number: JL10484‐48T), and CCL2 (catalogue number: JL20304‐48T).^42^

### 2.21 Co‐culture of microglia and neurons using a transwell system

Neuronal HT22 cells (catalogue number: MZ‐2157, Ningbo Mingzhou Biotechnology Co., Ltd.) and BV2 microglia from each group were singularly positioned in 24‐well dishes and Transwell inserts (.4 µm, Corning) for 24 h of co‐culture. Following the co‐culture, observation of morphological alterations in HT22 cells was conducted, and the cells were collected for further experiments.^43^

### 2.22 Cell counting kit‐8 (CCK‐8) analysis

The CCK‐8 (catalog number: CA1210, Beijing Solarbio Science & Technology Co., Ltd.) was employed to ascertain the viability of neurons. In brief, each well in a 96‐well plate received a combination of neurons along with 10 µL of CCK‐8 solution. Following incubation at 37°C, readings of absorbance at 450 nm were taken with a spectrophotometer (BioTek).^44^

### 2.23 TUNEL

At the cellular level, neuronal apoptosis was assessed using the Cell Meter TUNEL apoptosis assay kit (catalogue number: T2130, Beijing Solarbio Science & Technology Co., Ltd.). Specifically, the treatment of cells for fixation involved treating them with 4% paraformaldehyde for 15 min at 37°C, followed by permeabilization on ice for 2 min with a solution comprising .1% Triton X‐100 and .1% sodium citrate. The TUNEL reaction mixture underwent incubation at 37°C for a duration of 60 min, succeeded by counterstaining of cell nuclei with DAPI (catalogue number: C0065, Beijing Solarbio Science & Technology Co., Ltd.) to identify TUNEL‐positive cells. Calculation of the apoptotic rate (%) was based on the proportion of positive cells: apoptosis rate = number of apoptotic‐positive cells/total cell count × 100%.^45^

Brain tissue sections were deparaffinized and rehydrated at the tissue level, and the process of antigen retrieval involved the utilization of a sodium citrate buffer lasting 5 min. Then, inhibiting non‐specific antibody attachment was achieved by blocking the sections with 5% goat serum for 60 min. Overnight incubation was performed on the brain sections using the TUNEL reagent and rabbit anti‐NeuN antibody (1:100, ab177487, Abcam) at 4°C. Afterwards, they were exposed to a secondary antibody, Goat anti‐rabbit IgG H&L (Alexa Fluor 647; 1:500, ab150079, Abcam). After 1 h light‐protected incubation at standard room conditions, the sections underwent counterstaining utilizing the nuclear marker DAPI for 5 min in subdued light and analyzed for fluorescence intensity applying an Olympus BX51 fluorescence microscope (Olympus Corp) coupled with ImageJ imaging software.^46^

### 2.24 LDH release

Leakage of lactate dehydrogenase (LDH) into the cell culture medium indicates membrane integrity loss and cell death. After centrifuging and collecting the HT22 cell culture medium, an LDH cytotoxicity assay kit (C20301, Invitrogen) was utilized to assess LDH levels as per the manufacturer's stipulated guidelines.^47^

### 2.25 Grouping of CIRI mice

Adeno‐associated virus (AAV) encoding sh‐NC and sh‐E2F1 (catalogue number: A51217CN, ThermoFisher) was administered to Cx3cr‐Cre mice to facilitate microglia‐specific E2F1 knockdown. The efficient interfering sequence in mice E2F1, AAV‐E2F1 shRNA, was represented by the shRNA sequence 5′‐GCCAAGAAGTCCAAGAATCAT‐3′. A nontargeting control virus was constructed using the interfering sequence 5′‐GGTATTTAGTTGACACCTCAT‐3′. These viruses had a 3×10^12^ vg/mL titer and were delivered using stereotactic surgery. Briefly, under sedation and immobilization facilitated by a stereotactic apparatus provided by RWD Life Science, Cx3cr‐Cre mice underwent preparation for experimentation. The skull underwent drilling to create a hole with a burr, and the virus solution was infused into the brain tissue employing a motorized microinjector (Hamilton) at specific coordinates: hippocampal CA1 region (AP: −2.00 mm, ML: −1.55 mm, DV: −1.55 mm), cerebral cortex region (AP: 0.00 mm, ML: −2.05 mm, DV: −1.50 mm), and striatum region(AP: 0.00 mm, ML: −2.05 mm, DV: −3.50 mm), the administration involved a total of 500 nL injected over a period, delivering at a consistent pace of 50 nL/min. Implementation of the CIRI model occurred four weeks post‐injection, as detailed in the procedure.^44^

### 2.26 Hematoxylin and eosin (H&E) staining

Mouse brain tissue sections were deparaffinized in water after mounting on slides, followed by H&E staining per the directions outlined in the H&E staining kit (PT001, purchased from Shanghai Bogu Biotechnology Co., Ltd.). The primary procedures encompassed: hematoxylin staining for 10 min at RT, then rinsing in flowing water for 30–60 s; differentiation in 1% hydrochloric acid alcohol for 30 s, succeeded by washing and immersion for 5 min; eosin staining at RT for 1 min; dehydration in a range of alcohol concentrations (70%, 80%, 90%, 95%, and 100%) for 1 min each; clearing in xylene for 1 min, and twice in xylene I and II for 1 min each; mounting with neutral gum in a fume hood, and finally, capturing images of the morphological alterations in the brain tissues of each experimental group through the utilization of an optical microscope (BX50; Olympus Corp).

### 2.27 Immunohistochemistry

Following fixation in a 10% formalin solution, the mouse brain specimens experienced two rounds of xylene dewaxing, each lasting 10 min. Following rehydration in a graded ethanol series (100%, 95%, 75%, 50%) for 5 min each, the tissues were processed with .01 mol/L citrate buffer, microwaved for 20 min, incubated at RT with 1 drop of H_2_O_2_ for 10 min, washed, incubated with one drop of normal goat serum for 5 min, kept overnight at 4°C with primary antibody targeting the microglial cell marker Iba‐1 (1:2000, ab178846, Abcam). Subsequently, tissues underwent a 1 h incubation period at 37°C, then were exposed to biotinylated goat anti‐rabbit secondary antibody (1:500, ab150077, Abcam) for 30 min at the same temperature. Next, the freshly prepared DAB chromogen solution (DA1015, Beijing Soleibao Technology Co., Ltd.) was added for 1–2 min. Hematoxylin (G1080, Beijing Soleibao Technology Co., Ltd.) was applied as a counterstain for 1 min. Subsequent steps involved dehydration, clearing, and covering with a neutral mounting medium. Five random high‐power fields were chosen for microscopic examination.^48^

### 2.28 TTC staining

Following the CIRI procedure, the euthanasia of mice was performed, and an ischemic infarction assessment was conducted with TTC staining. In brief, the mouse brains were rapidly extracted and flash‐frozen at −20°C for 10 min. Prepared brain tissue sections were exposed to 1% TTC (Sigma‐Aldrich) staining for a period of 30 min at 37°C. Subsequently, sections of the components were partly soaked and anchored in a 4% PFA solution. ImageJ application facilitated the evaluation of the ischemic infarct area in different segments. The ischemic infarct areas were summed as described below: Infarct area (%) = (contralateral area − ipsilateral noninfarct area)/contralateral area × 100%.^44^, ^47^

### 2.29 Morris water maze test

The Morris water maze (MWM) test is used for appraising spatial learning and memory capabilities. Simply put, the water maze encompasses a cylindrical water tank with a diameter of 120 cm, filled with opaque water that is 60 cm high, with a temperature of 22 ± 2°C, and an approximately 6 cm‐wide circular platform is positioned 1 cm below the level of the water. The tank is adorned with a variety of shapes on its walls to serve as spatial cues for reference. An advanced digital monitoring system (Neuron Information Technology) is positioned overhead to monitor and document the navigating routes within the water maze. The test is conducted daily at the same time as mice. During the hidden platform training phase, the training regimen for mice involves 6 sequential days on an underwater platform, including four trials each day. During every trial, the animals are set free at the edge of the tank and allowed to locate and ascend the concealed platform within a designated 60 s examination timeframe. If an animal fails to locate the platform within the allocated timeframe, it is gently directed towards the platform and given 15 s to enhance cognitive processes and spatial memory development. Probe trials are conducted 24 h posttraining. During these probe trials, the platform is eliminated, and the behaviours of the mice are recorded for 60 s. Factors including the period allocated in the target sector, time spent to locate the platform, distance traversed to approach the target, and the digital marking of the complete platform are autonomously registered for future scrutiny.^44^, ^47^

### 2.30 Novel object recognition

Before the experiment, laboratory mice underwent a 2‐day acclimation period, with 30 min sessions on consecutive days. At the commencement of the study, each mouse was introduced into an open field apparatus (50 cm × 50 cm × 50 cm) containing two indistinguishable items (familiar objects) positioned together on a specific area within the open field container, where they were given the freedom to investigate the items for a duration of 5 min. After a lapse of 1 h, a different object took the position of one of the familiar objects, and 5 min exploration period was allotted to the animals. The exploration timeframe of objects, whether recognizable or completely new, was registered through a digital video tracking system (Neuron Information Technology). Between each trial, olfactory cues were eliminated from the chambers and items by cleaning them with a 75% ethanol solution. The consequences were assessed through interactions involving the object (excluding solely the tail) or being in front of the object (distance < 2 cm). In the evaluation of cognitive function associated with CA1, the discrimination index was computed by contrasting the duration spent on investigating the unfamiliar target to the total exploration time, with a more pronounced inclination towards the unfamiliar item suggesting undamaged spatial memory recognition.^44^, ^47^

### 2.31 Analytical statistics

Utilization of GraphPad Prism 9 software from GraphPad Software, Inc. and the R programming language facilitated the statistical analysis in this study. Continuous data are displayed as Mean ± SD. The comparison of two groups characterized by a normal distribution involved the application of either the unpaired student's *t*‐test or the Wilcoxon test, while for comparing data among multiple groups, one‐way ANOVA with Tukey's post hoc test was executed. Demonstrating variations required establishing statistical significance at *p* < .05.

## 3 RESULTS

### 3.1 Mitochondrial‐associated genes in the key role and regulatory network analysis of ischemic CIRI

**FIGURE 1:**
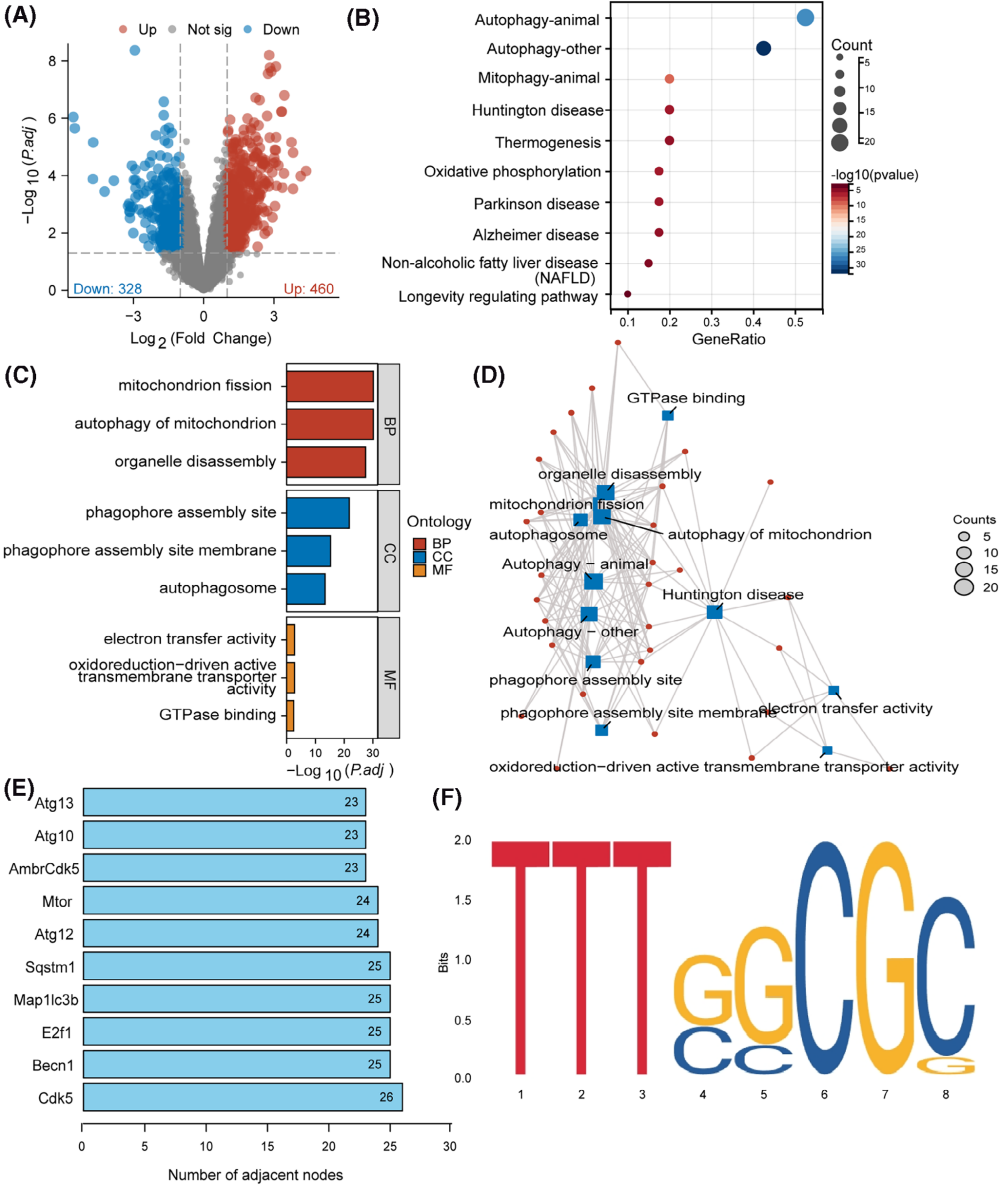
Potential molecular mechanisms influencing CIRI. (A) Volcano plot of DEGs in the transcriptome sequencing dataset of CIRI mouse brain tissue, red dots represent upregulated genes in CIRI mouse brain tissue and blue dots represent downregulated genes. (B) KEGG enrichment analysis of the 25 most significantly upregulated and downregulated genes in CIRI mouse brain tissue. (C) Bar graph of functional enrichment analysis of the 25 most significantly upregulated and downregulated genes in CIRI mouse brain tissue. (D) Network diagram of functional enrichment analysis, blue dots represent enriched functional names, the size of the dots represents the number of enriched genes, larger dots indicate more enriched genes, and red dots represent the enriched genes. (E) Bar graph of core gene selection. (F) Transcription binding site of E2F1.

Ischemia and hypoxia exert a profound impact on the brain's susceptibility. Managing ischemic cerebrovascular disorders often involves the use of mechanical thrombectomy and the intravenous introduction of tissue plasminogen activator to rapidly reestablish blood flow in brain areas affected by ischemia. Nevertheless, the subsequent impairment impacting brain operation following reperfusion, referred to as CIRI, poses a difficult problem to tackle.^49^ To uncover the molecular mechanisms influencing CIRI, we first successfully established a CIRI mouse model (Figure S1A,C). Subsequently, based on transcriptome sequencing, we conducted molecular‐level differential analysis on brain tissue from the CIRI mouse model. The data depicted in Figure 1A suggest that relative to the Sham group, the brain tissue of CIRI mice exhibited upregulation of 460 genes and downregulation of 328 genes. Next, we selected the 25 most significantly upregulated and downregulated genes for GO and KEGG analysis, which highlighted significant enrichment in pathways related to mitophagy, oxidative phosphorylation, and neurodegenerative diseases such as “Autophagy—animal”, “Oxidative phosphorylation”, “Huntington's disease”, and “Parkinson's disease” (Figure 1B). These genes were primarily enriched in biological functions associated with mitochondrial dysfunction, such as “mitochondrion fission”, “autophagy of mitochondrion”, and “autophagosome” (Figure 1C,D). Previous studies have confirmed the close association between mitochondrial dysfunction and CIRI, implicating it as a pivotal target area for neuronal cell death postischemia.^50^

Wed an in‐depth analysis of 50 candidate genes in the core gene network. The analysis of the key genes playing essential roles in regulating the functionless above revealed (Figure 1E) that CDK5, encoded by the CDK5 gene, occupies a central position in the network. This discovery highlights the central role of CDK5 in the entire regulatory network, indicating its potential as a significant molecular driver influencing mitochondrial‐related biological functions. The transcription factor E2F1, encoded by the E2F1 gene, also emerged as another key gene of interest. Analysis of online databases identified a binding site TTTGGCGG for E2F1 on the CDK5 promoter region (Figure 1F), indicating that E2F1 may be implicated in the transcriptional regulation of CDK5. Furthermore, literature studies have indicated that DRP1, a pivotal regulatory protein in mitochondrial fission, is integral to the formation of mitochondrial dysfunction via phosphorylation at its Ser616 site and dephosphorylation at Ser637.^51^ Building on this, we hypothesize that the kinase CDK5 may intervene in mitochondrial dysfunction during CIRI by influencing the phosphorylation status of DRP1.

### 3.2 scRNA‐seq reveals the significance of E2F1 and CDK5 in microglia within CIRI mouse brain tissue

**FIGURE 2:**
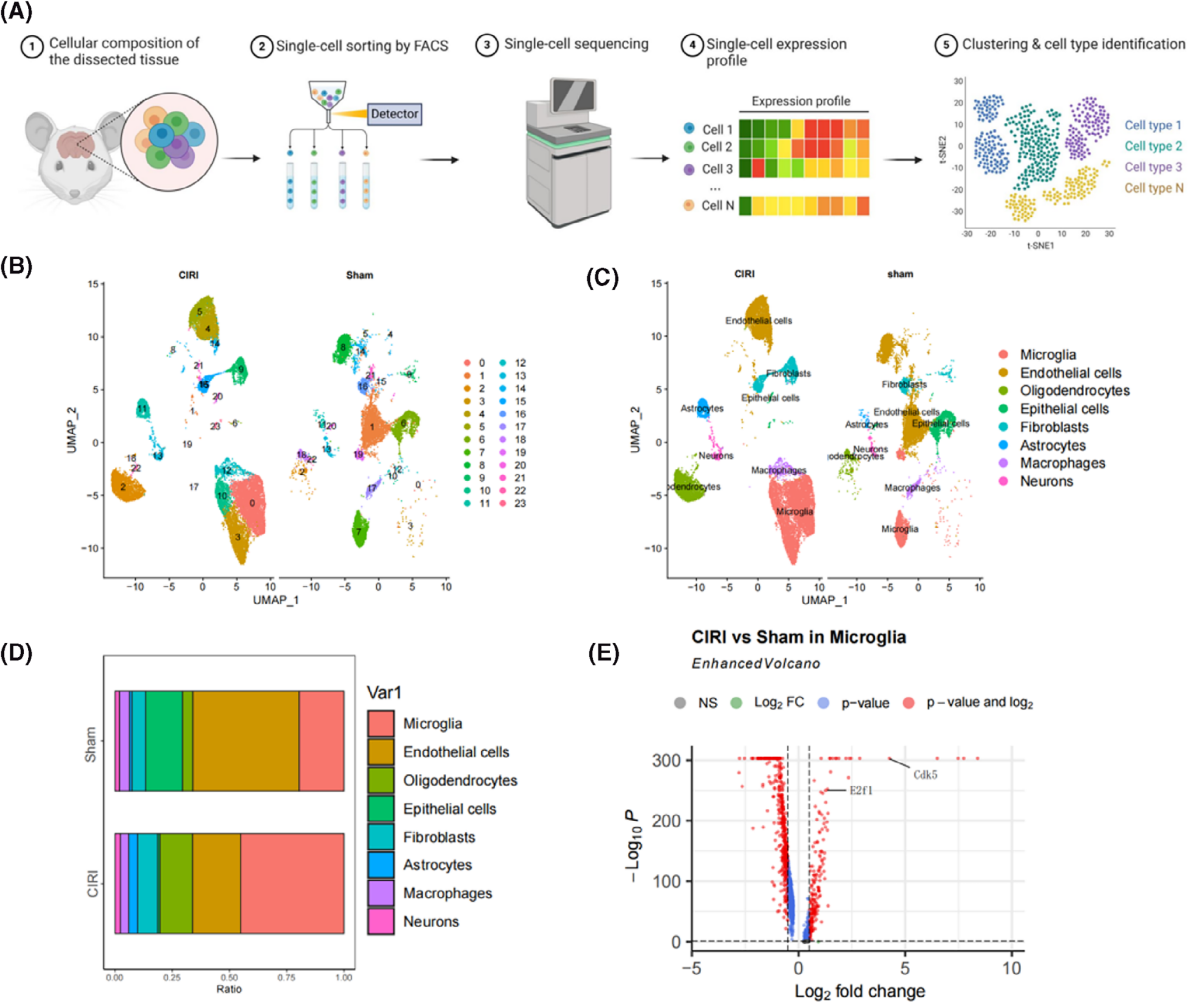
Cellular heterogeneity analysis in the brain tissue of CIRI mice. (A) Flowchart of single‐cell sequencing in brain tissue (created with BioRender.com); (B) UMAP clustering of cells in CIRI mouse brain tissue compared to Sham mouse brain tissue into 24‐cell clusters. (C) Annotation of the 24 cell clusters into eight cell types. (D) Distribution of microglia proportions in each sample; (E) Analysis of DEGs in microglia within brain tissue.

The exploration of gene expression patterns at a single‐cell level provided insights into the cellular heterogeneity and complexity in CIRI mouse brain tissue. To achieve this, we established a CIRI mouse model and collected brain tissue samples for scRNA‐seq analysis (Figure 2A) while employing healthy mouse brain tissue as a control group.

The R package “Seurat” was utilized for scRNA‐seq data analysis. Once the scRNA‐seq data underwent quality control measures and standardization, a correlation of *r* = −.31 was noted between nCount and percent.mt and a correlation of *r* = .9 was found between nCount and nFeature (Figure S2A), presenting favourable cell characteristics subsequent to filtering. Later on, the operation of RunPCA was applied to decrease the dimensionality of PCA concerning the most variable 2000 genes (Figure S2B), revealing the absence of evident batch discrepancies across tissue specimens (Figure S2C). The visualization of the leading 40 PCs was achieved through the utilization of the JackStrawPlot function (Figure S2D). Here, our demonstration focuses on the key genetic components found within the first six PCs (Figure S2E).

Subsequently, utilizing UMAP analysis, we investigated the cellular heterogeneity in brain tissue samples from Sham and CIRI mice, revealing (Figure 2B) a total clustering of 24 cell clusters. Through annotation of the cell clusters with known cell marker genes, we identified eight cell types (Figure 2C; Figure S3): microglia, endothelial cells, oligodendrocytes, epithelial cells, fibroblasts, astrocytes, macrophages, and neurons. Notably, the proportion of Microglia substantially elevated in the brain tissue samples from CIRI mice (Figure 2D), suggesting a potentially critical role in CIRI. Consequently, we proceeded with further analysis of the gene expression profiles of microglia within brain tissue from Sham and CIRI mice, revealing (Figure 2E) a significant upregulation of E2F1 and CDK5 expression in microglia. The expression of E2F1 and CDK5 in astrocytes and neurons showed no significant changes (Table S2).

**FIGURE 3:**
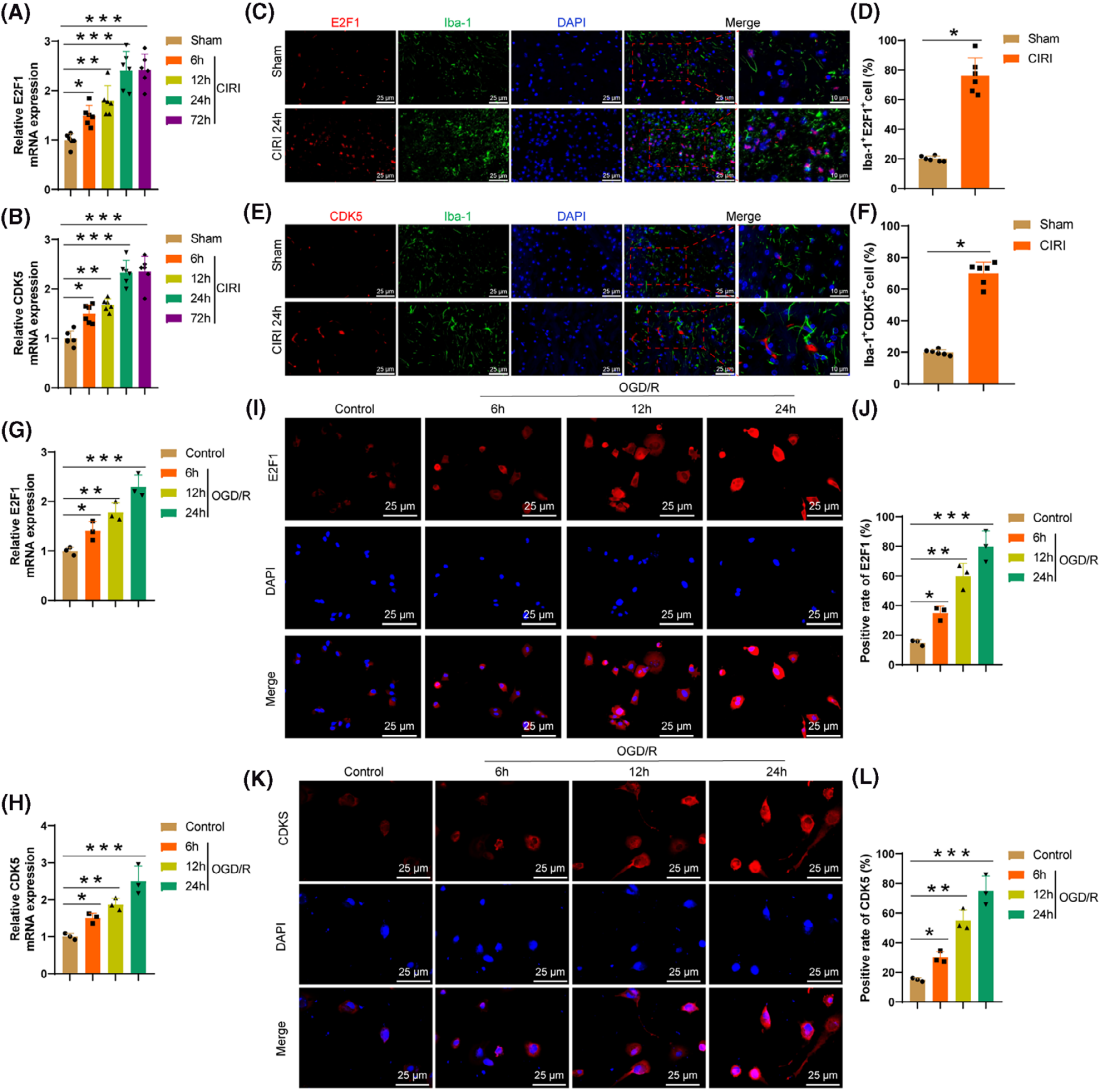
Expression levels of E2F1 and CDK5 in microglia after CIRI. (A, B) RT‐qPCR experiments assessing the expression levels of E2F1 and CDK5 in the brain tissue of CIRI mice. (C–F) Immunofluorescence experiments detected the expression levels of E2F1 (C, D) and CDK5 (E, F) in the brain tissues of mice 24 h after CIRI. (G, H) RT‐qPCR experiments measuring the expression levels of E2F1 and CDK5 in microglia after OGD/R, scale bars = 25 µm. (I, L) Immunofluorescence experiments investigating the expression levels of E2F1 (I, J) and CDK5 (K, L) in microglia after OGD/R. Scale bars = 25 µm. Each group consisted of six mice, and all cellular experiments were repeated three times. *p < .05, p** < .01, ***p < 0.001.

The RT‐qPCR results illustrate (Figure 3A,B) a considerable enhancement in the levels of E2F1 and CDK5 expression in brain tissue at 6 h post‐CIRI, peaking at 24 h. Immunofluorescence experiments reveal (Figure 3C–F) a notable upregulation of E2F1 (Figure 3C,D) and CDK5 (Figure 3E,F) expression in microglia within the brain tissue at 24 h post‐CIRI, with Iba‐1 as a microglia marker. BV2 cells were subjected to OGD/R to simulate CIRI conditions. The RT‐qPCR findings (Figure 3G,H) demonstrate a gradual elevation in the expression levels of E2F1 and CDK5 in microglia with prolonged OGD/R exposure. Immunofluorescence analysis confirms these results (Figure 3I–L).

Research indicates that post‐CIRI, activated microglia can induce brain tissue inflammation by releasing inflammatory factors.^52^ The upregulation of E2F1 and CDK5 may reflect the complex roles played by microglia in response to CIRI. Therefore, it is speculated that E2F1 and CDK5 could serve as crucial regulatory factors in the aetiology of CIRI and potential targets for upcoming therapeutic approaches.

### 3.3 Molecular mechanism study of transcription factor E2F1 regulating the transcriptional activity of the CDK5 gene

**FIGURE 4:**
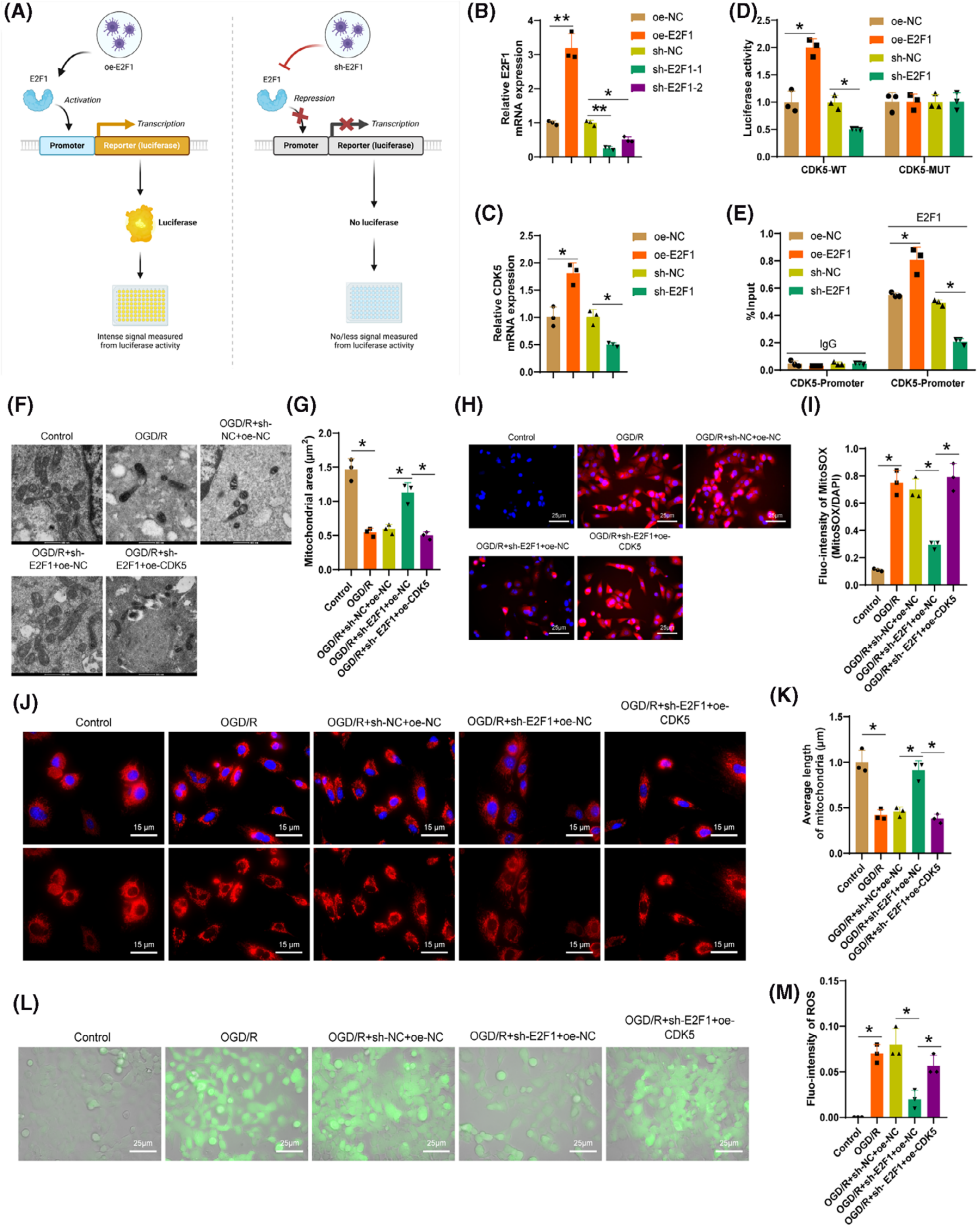
Transcriptional regulation of CDK5 by E2F1 in microglia. (A) Schematic diagram of the dual‐luciferase reporter gene experiment (Created with BioRender.com). (B) RT‐qPCR detection of E2F1 expression levels in cells after overexpression or silencing of E2F1. (C) RT‐qPCR detection of CDK5 expression levels in cells after overexpression or silencing of E2F1. (D) Dual‐luciferase assay to detect the effect of E2F1 on CDK5 promoter transcriptional activity: based on lentivirus‐mediated silencing and overexpression of E2F1 in 293T cells (oe‐NC, oe‐E2F1, sh‐NC, sh‐E2F1), co‐transfected with the dual‐luciferase reporter gene vector containing the CDK5 promoter sequence and its binding site mutant, luciferase activity was measured 48 h posttransfection and normalized to Renilla. (E) ChIP experiment to detect the enrichment of E2F1 on the CDK5 promoter. (F, G) TEM image showing mitochondrial morphology in oligodendrocytes. Scale bar = 500 nm. (H, I) Fluorescence images and related quantitative data showing Mito‐ROS MitoSOX fluorescence in oligodendrocytes of each group. Scale bar = 25 µm. (J, K) Immunofluorescence detection of mitochondrial morphology in oligodendrocytes of each group, red marks mitochondria, blue marks nuclei. Scale bar = 15 µm. (L, M) DCFH‐DA staining to detect ROS production in oligodendrocytes of each group. Scale bar = 25 µm. All cellular experiments were conducted thrice, *p < .05, **p < .01.

To further investigate the regulatory role of the transcription factor E2F1 on the transcriptional activity of the CDK5 gene, we conducted experiments using lentiviruses to silence or overexpress E2F1 in 293T cells. These cells were then grouped accordingly and assessed through dual‐luciferase reporter gene assays (Figure 4A). The RT‐qPCR results (Figure 4B) unveiled a considerable enhancement in the levels of E2F1 expression in cells following E2F1 overexpression and a noticeable decrease in E2F1 expression upon E2F1 silencing, with sh‐E2F1‐1 demonstrating the most effective silencing for subsequent experiments. Furthermore, the expression levels of CDK5 significantly increased after E2F1 overexpression and notably decreased following E2F1 silencing (Figure 4C). The dual‐luciferase reporter assay data (Figure 4D) indicated a substantial elevation in CDK5 promoter activity after E2F1 overexpression and a significant decrease upon E2F1 silencing, while the mutant groups showed no significant changes. ChIP experiments (Figure 4E) revealed a pronounced surge in E2F1 enrichment on the CDK5 promoter following E2F1 overexpression and a substantial decrease in E2F1 enrichment upon E2F1 silencing. It is indicated by the findings that E2F1 can promote CDK5 transcription in microglia by binding to the CDK5 promoter region.

To delve deeper into the impact and mechanisms of E2F1/CDK5 proteins on mitochondrial dysfunction in brain CIRI, we knocked out E2F1 in the primary astrocyte OGD/R model and studied the effect of CDK5 overexpression on mitochondrial morphology and ROS accumulation. TEM images (Figure 4F,G) showed that the knockout of E2F1 reduced the number of structurally disordered mitochondria, and increased their area and perimeter, preserving mitochondrial structural integrity. However, upon CDK5 overexpression, mitochondria exhibited phenotypes consistent with OGD/R‐induced changes. Confocal microscopy images (Figure 4J,K) revealed a considerable drop in filamentous mitochondria and a rise in punctate mitochondria with severe mitochondrial fragmentation in astrocytes post‐OGD/R. Knockout of E2F1 reversed this process, but the effect was abolished when CDK5 was overexpressed, suggesting that E2F1 may induce astrocytic mitochondrial fission by promoting CDK5 expression.

Following OGD/R, trends in Mito‐ROS were assessed by applying MitoSOX, showing an elevation in Mito‐ROS levels post‐OGD/R. Knockout of E2F1 led to a notable drop in ROS (Mito‐ROS), whereas CDK5 overexpression reversed the declining trend caused by E2F1 knockout (Figure 4H,I). Furthermore, an increase in fluorescence intensity of DCFH‐DA in astrocytes indicated ROS accumulation, which was reversed by E2F1 knockout but restored by CDK5 overexpression (Figure 4L,M), providing additional evidence that E2F1 may promote ROS accumulation in astrocytic mitochondria through upregulation of CDK5.

### 3.4 Mechanisms of CDK5‐mediated mitochondrial fission and ROS accumulation in the CIRI model

**FIGURE 5:**
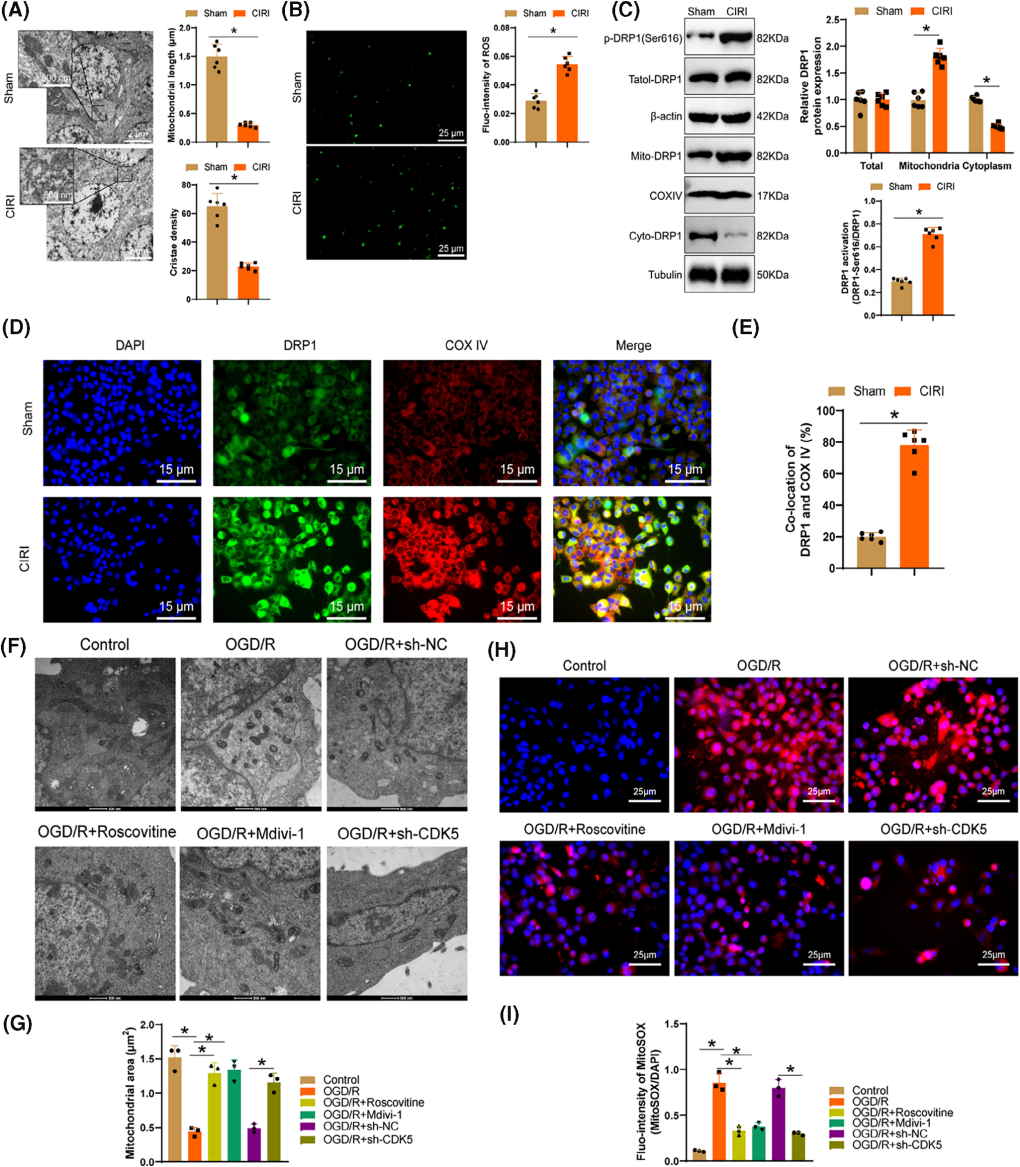
CDK5 regulation of DRP1 phosphorylation affects microglia mitochondrial fission and ROS accumulation. (A) TEM image displaying mitochondria in the cerebral cortex post‐CIRI along with quantitative data on mitochondrial length and cristae density per group. Scale bars = 2 µm/500 nm. (B) DCFH‐DA staining assessing ROS production in the cerebral cortex post‐CIRI. Scale bar = 25 µm. (C) Western blot determining the activation status of DRP1, with β‐actin, COX IV, and Tubulin serving as internal references for total, mitochondrial, and cytosolic fractions, respectively. (D, E) Immunofluorescence detecting the co‐localization of DRP1 and mitochondrial marker protein COX IV. Scale bar = 15 µm; (F, G) TEM image featuring mitochondrial morphology in microglia. Scale bar = 500 nm. (H, I) Fluorescence images and corresponding quantitative data showing MitoSOX fluorescence in microglia of each group. Scale bar = 25 µm. Each group comprised six mice, and all cellular experiments were repeated three times. *p < 0.05.

Studies have reported that releasing fragmented and dysfunctional microglial mitochondria into the neuronal environment can lead to the propagation of damage,^53^ and CDK5 can mediate the phosphorylation of DRP1.^41^ Based on the aforementioned studies, to ascertain whether CDK5 in CIRI can mediate mitochondrial fission by regulating the phosphorylation of DRP1, the effects of CDK5 overexpression and knockout on DRP1 phosphorylation were initially validated at the cellular level, followed by verification experiments in the CIRI mouse model. TEM images of CIRI model mice revealed a significant increase in mitochondrial fragmentation and cristae vacuolization in the cerebral cortex after CIRI (Figure 5A). In addition, the ROS levels in the cerebral cortex significantly increased (Figure 5B), indicating that CIRI can lead to mitochondrial fission and ROS accumulation. Western blot experiments demonstrated (Figure 5C) that post‐CIRI, DRP1 mainly translocates to and accumulates on mitochondria through activation at DRP1‐Ser616. Through immunofluorescence imaging (Figure 5D,E), a substantial elevation was detected in the colocalization of DRP1 and the mitochondrial marker protein COX IV, further confirming the accumulation of DRP1 on mitochondria after CIRI.

Subsequently, we categorized the primary astrocytes in the OGD/R model by knocking down CDK5 or utilizing the CDK5 inhibitor Roscovitine and the DRP1 inhibitor Mdivi‐1. The results of western blot experiments (Figure S4A,B) illustrated a remarkable surge in the phosphorylation levels of DRP1‐Ser616 in cells after CDK5 knockdown or treatment with Roscovitine or Mdivi‐1, along with a marked reduction of DRP1 in mitochondria. Confocal images (Figure S4C,D) showed a noticeable decrease in linear mitochondria and increased punctate mitochondria, along with severe mitochondrial fragmentation in primary astrocytes post‐OGD/R, which could be reversed by Roscovitine, Mdivi‐1, and CDK5 knockdown. TEM images (Figure 5F,G) demonstrated that CDK5 knockdown or treatment with Roscovitine and Mdivi‐1 post‐OGD/R reduced the number of structurally disorganized mitochondria while increasing mitochondrial area and perimeter, thereby preserving mitochondrial structure and morphology. These results suggest that CDK5 possibly induces mitochondrial fission in primary astrocytes by promoting the phosphorylation of DRP1 at the Ser616 site.

Following OGD/R, an evident enhancement in DCFH‐DA fluorescence intensity was observed in microglial cells, pointing to the presence of accumulated ROS inside the cells. Deletion of CDK5 or the addition of Roscovitine and Mdivi‐1 reversed this process (Figure S4E,F). Additionally, analysis of Mito‐ROS utilizing MitoSOX revealed a trend in line with the changes in total ROS levels (Figure 5H,I), further confirming that CDK5 may potentially promote the accumulation of ROS in microglial cell mitochondria by enhancing the phosphorylation of DRP1 at the Ser616 site.

### 3.5 Regulation of DRP1 activity about microglial mitophagy and function

Excessive accumulation of ROS can trigger cellular stress, leading to mitochondrial impairment.^54^ Mitophagy, a process of clearing damaged mitochondria, is essential, as inhibition of autophagy can result in mitochondrial dysfunction.^55^ Previous research has indicated that CDK5 may induce the accumulation of ROS in microglial mitochondria by promoting phosphorylation at the Ser616 site of DRP1. Moving forward, we aim to investigate the impact of activated DRP1 on mitophagy.

**FIGURE 6:**
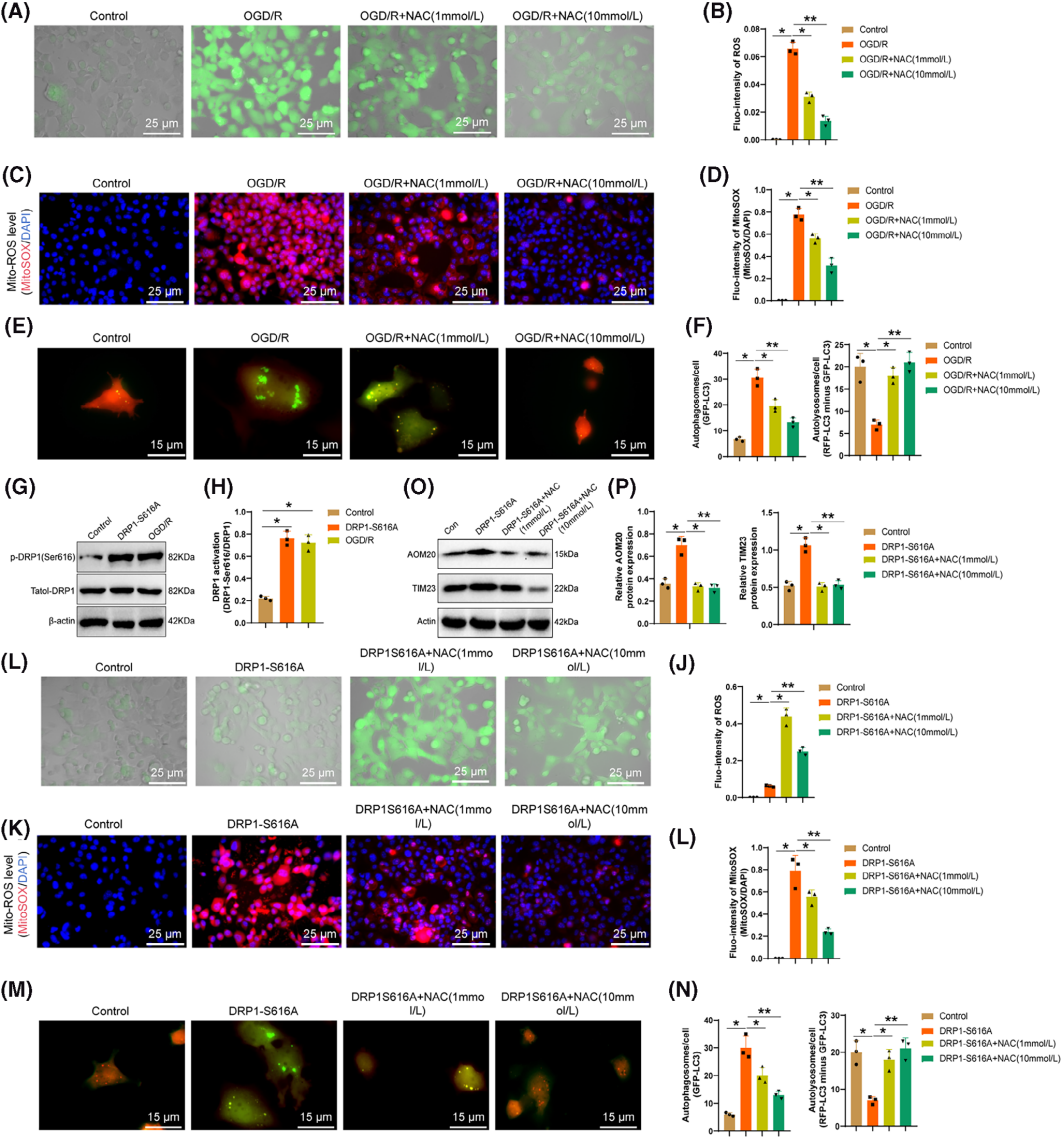
Impact of activated DRP1 on mitophagy. (A, B) DCFH‐DA staining to assess ROS production in microglia of each group. Scale bar = 25 µm. (C, D) Fluorescence images and corresponding quantitative data displaying Mito‐ROS MitoSOX fluorescence in microglia of each group. Scale bar = 25 µm. (E, F) Observation of autophagic flux in microglia using the mRFP‐GFP‐LC3 reporter system. Scale bar = 15 µm. (G, H) Western blot analysis of DRP1 activation status. (I, J) DCFH‐DA staining to detect ROS production in microglia of each group. Scale bar = 25 µm. (K, L) Quantification of Mito‐ROS MitoSOX fluorescence in microglia of each group through fluorescence images. Scale bar = 25 µm. (M, N) Evaluation of autophagic flux in microglia utilizing the mRFP‐GFP‐LC3 reporter system. Scale bar = 15 µm. (O, P) Western blot results and quantification of mitochondrial‐specific proteins. All cellular experiments were repeated three times. *p < .05, **p < .01.

To investigate this, we treated OGD/R‐exposed microglia with the ROS scavenger NAC. Our findings (Figure 6A,D) revealed a substantial and dosage‐related reduction in the fluorescence intensity of DCFH‐DA in microglia post‐NAC treatment compared to the OGD/R group, confirming the ability of NAC to decrease ROS accumulation within cells, with Mito‐ROS levels following a similar trend. Using the mRFP‐GFP‐LC3 reporter system to observe autophagic flux in microglia, it was observed (Figure 6E,F) that in the normal control group, autophagosomes were efficiently engulfed by lysosomes to generate autolysosomes, as indicated by the high fluorescence intensity of “RFP‐LC3 minus GFP‐LC3,” suggesting smooth autophagic flux. Conversely, in OGD/R‐treated microglia, excessive autophagic bodies (GFP‐LC3) remained unconsumed in the acidic lysosomal environment, as shown by the reduced fluorescence intensity of “RFP‐LC3 minus GFP‐LC3,” signifying incomplete autophagosome‐lysosome conversion following OGD/R. However, treatment with NAC showed a significant dose‐dependent improvement in converting autophagic bodies into autolysosomes, indicating that ROS accumulation in microglia post‐OGD/R could hinder mitophagy.

Furthermore, we enhanced DRP1 activity in microglia by utilizing the DRP1‐S616A mutation (Figure 6G,H) and observed (Figure 6I–L) that in contrast with the normal control group, microglia treated with DRP1‐S616A exhibited a prominent escalation in ROS levels, which were subsequently reduced upon NAC treatment to mitigate cellular ROS accumulation. Evaluation of autophagic flux (Figure 6 M,N) in microglia treated with DRP1‐S616A indicated that excessive autophagic bodies (GFP‐LC3) remained uncleared in the acidic lysosomal environment. The diminished fluorescence intensity of “RFP‐LC3 minus GFP‐LC3” suggested incomplete conversion of autophagic bodies into autolysosomes post DRP1‐S616A treatment, a process that was notably improved in a dose‐dependent manner with NAC treatment. This indicates that activated DRP1 may inhibit the conversion of autophagosomes into autolysosomes by promoting microglial ROS accumulation, leading to impaired mitophagy and subsequent mitochondrial dysfunction.

Furthermore, the relationship between activated DRP1 and mitochondrial autophagy was investigated by evaluating the degradation of mitochondrial proteins through Western blot analysis. A comparison of the changes in the mitochondrial outer membrane protein TOM20 and the inner membrane protein TIM23 in DRP1‐S616A indicated that as opposed to the control group, a surge in mitochondrial protein expression following DRP1‐S616A treatment suggested the inhibition of mitochondrial autophagy. However, after treatment with NAC, autophagy occurred (Figure 6O,P).

### 3.6 Activated DRP1 aggravates the inflammatory process in CIRI brain tissue by promoting microglial activation

**FIGURE 7:**
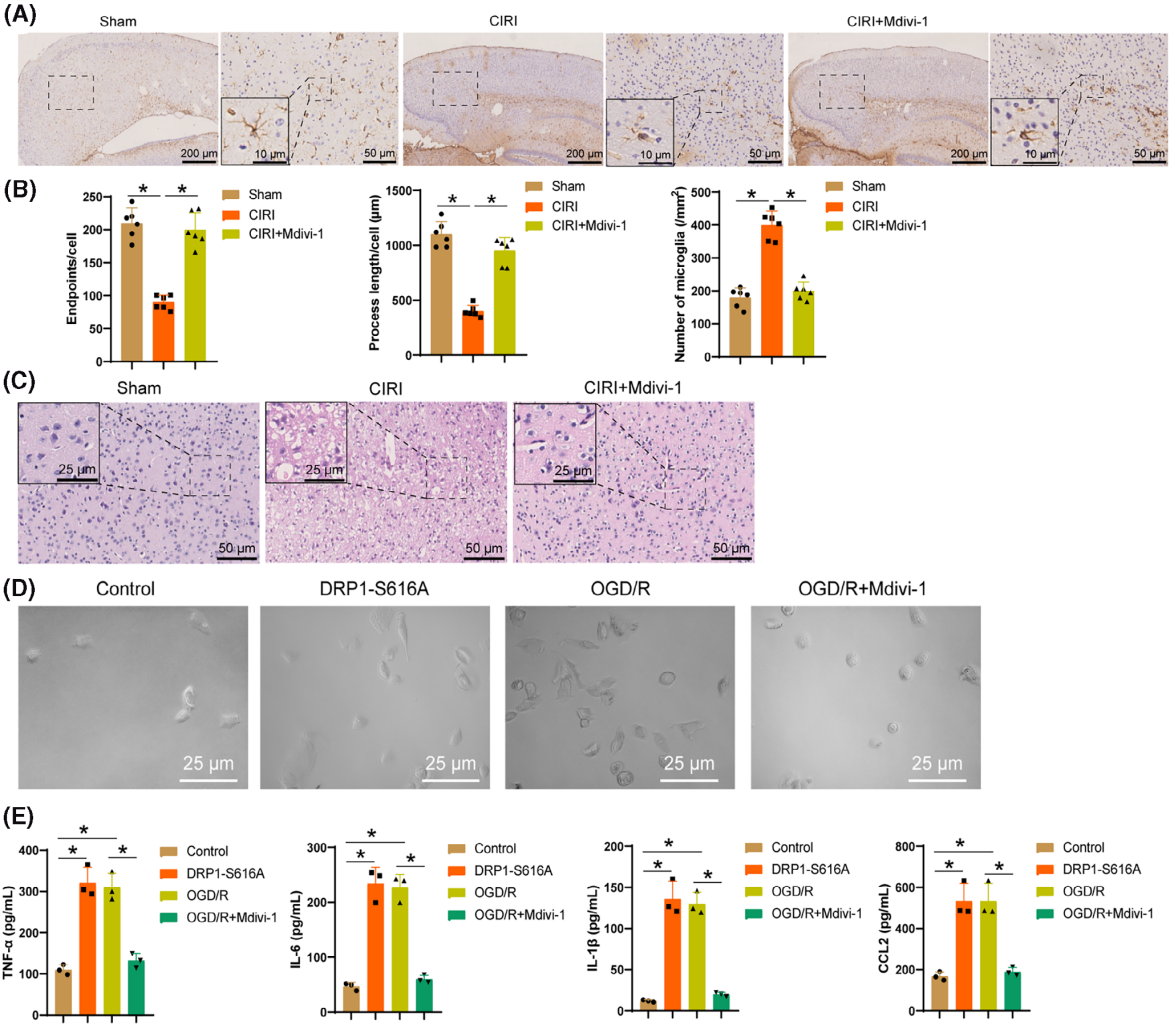
Impact of activated DRP1 on microglia activation. (A) Immunohistochemical staining of microglia marker Iba‐1 in brain tissue, Scale bar = 200/50/10 µm. (B) Endpoint/branch length/density of microglia in each group. (C) H&E staining of the cerebral cortex post‐CIRI, scale bar = 50/25 µm. (D) Cellular body morphological changes in microglia of each group. Scale bar = 25 µm. (E) ELISA analysis of secretion levels of inflammatory factors TNF‐α, IL‐6, IL‐1β, and CCL2 in microglia of each group. Each group consist of six mice, and all cellular experiments were repeated three times. *p < .05.

Mitochondrial dysfunction can activate microglia, triggering tissue inflammatory responses.^56^ To investigate the impact of activated DRP1 on microglial activation, we initially treated CIRI mouse models with the DRP1 inhibitor Mdivi‐1. Immunohistochemical staining of microglial marker Iba‐1 revealed (Figure 7A) that post‐CIRI, microglia in the cerebral cortex transitioned from a resting state to an activated phenotype, characterized by enhanced branching and hypertrophic morphology, a response that Mdivi‐1 reversed. Utilizing FIJI software and Sholl analysis (Figure 7B), we noted a drop in the quantity of endpoints and branch length per microglia following CIRI, along with a noteworthy enhancement in Iba‐1 immunostaining density in the brain, all of which were reversed by Mdivi‐1 treatment. H&E staining results (Figure 7C) demonstrated the reversal of significant inflammation infiltration in the brain tissues of CIRI mice by Mdivi‐1, suggesting its potential to facilitate the inflammatory processes in CIRI brain tissues.

Further research in microglia revealed that following DRP1‐S616A expression and OGD/R treatment, the cell bodies of microglia significantly enlarged (Figure 7D), accompanied by elevated secretion of inflammatory mediators TNF‐α, IL‐6, IL‐1β, and CCL2 (Figure 7E). This indicated a transition of microglia from a quiescent state to an activated state, a process that was mitigated by Mdivi‐1. In conclusion, the activation of DRP1 exacerbates the inflammatory process in CIRI brain tissue by promoting microglial activation.

### 3.7 Investigation of the molecular mechanisms of neuronal toxicity regulation by DRP1 and E2F1/CDK5 in microglia

Research has shown excessive microglia activation can damage neurons.^57^ A co‐culture system of microglia and neurons was prepared to elucidate the potential impact of DRP1 in microglia on neurons (Figure S5A). Experimental findings revealed that after treatment of microglia with DRP1‐S616A and OGD/R, the co‐cultured neurons exhibited degenerative morphological changes such as cell shrinkage and axon disappearance or shortening (Figure S5B). Additionally, the viability of neurons significantly decreased (Figure S5C), which was reversible by Mdivi‐1. Tunel staining and LDH assays (Figure S5D–F) indicated that microglia treated with DRP1‐S616A and OGD/R promoted neuronal apoptosis and LDH release, which Mdivi‐1 attenuated. These results suggest that inhibiting DRP1 can ameliorate the toxic effects of proinflammatory microglia on neurons.

**FIGURE 8:**
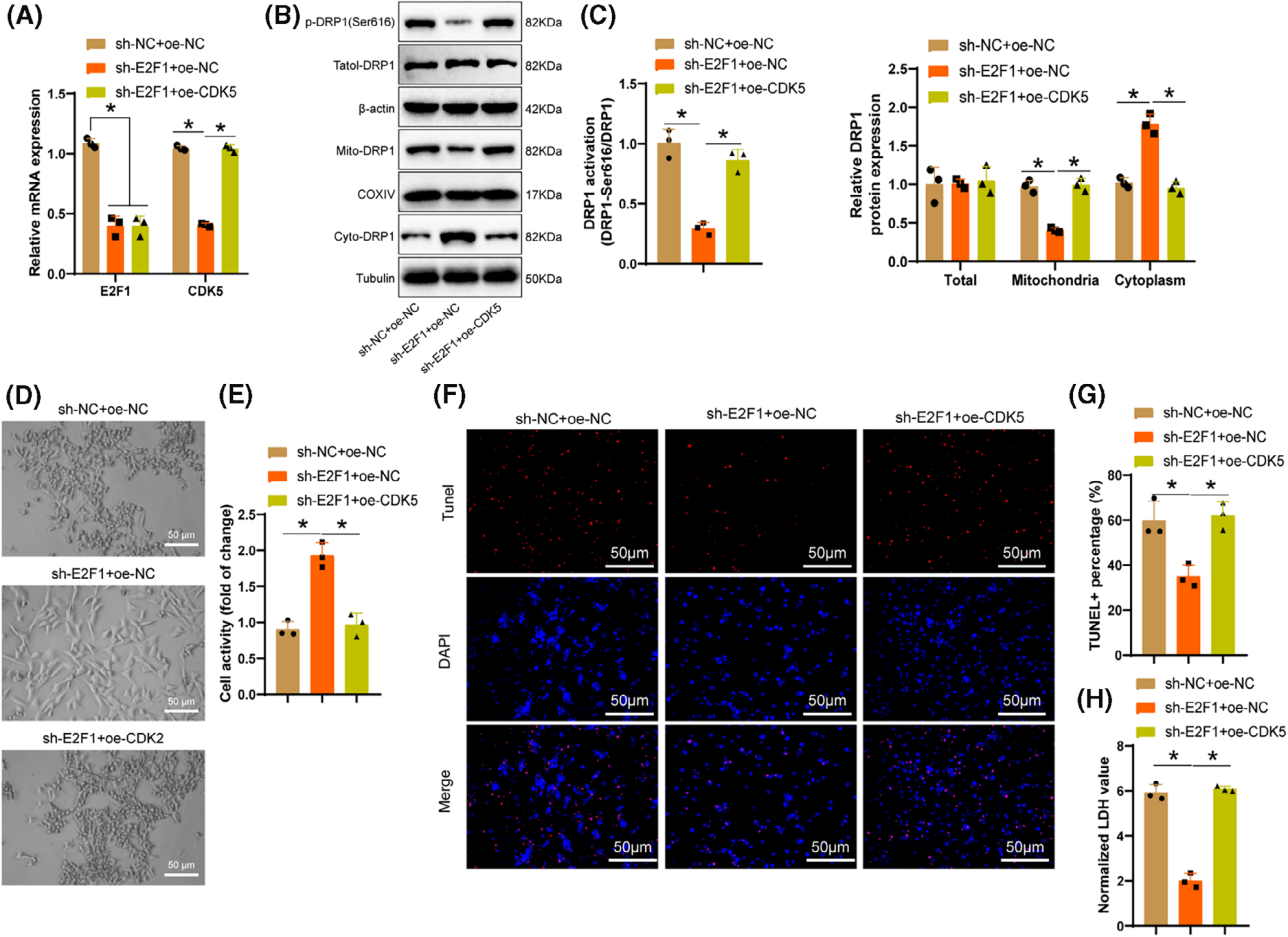
Toxic effects of E2F1/CDK5 regulating DRP1 on neurons. (A) RT‐qPCR analysis of E2F1 and CDK5 expression levels in cells. (B, C) Western blot examining the activation status of DRP1, with β‐actin, COX IV, and Tubulin used as internal references for total, mitochondrial, and cytosolic fractions. (D) Morphological changes in neurons in each group. Scale bar = 25 µm. (E) CCK‐8 assay measuring the proliferative activity of neurons in each group. (F, G) Tunel staining assessing apoptosis in neurons of each group. Scale bar = 50 µm. (H) Quantification of LDH release indicating neuronal cell death. All cellular experiments were conducted thrice. *p < .05.

To further investigate the toxic effects of E2F1/CDK5 on neurons through the regulation of DRP1, OGD/R‐treated cells were subdivided. RT‐qPCR and Western Blot results (Figure 8A–C) illustrated that silencing E2F1 resulted in reduced expression levels of E2F1 and CDK5 in microglia, leading to a significant decrease in phosphorylation of DRP1 at the Ser616 site and the expression of DRP1 in mitochondria; overexpression of CDK5 reversed these effects. Functional assessments of neurons revealed that silencing E2F1 improved the degenerative morphology of co‐cultured neurons (Figure 8D), significantly increased neuronal activity (Figure 8E), and inhibited neuronal apoptosis and LDH release (Figure 8F–H), effects that were reversible by CDK5 overexpression. These findings indicate that silencing E2F1 alleviates the toxicity of microglia on neurons, whereas overexpression of CDK5 can reverse this effect.

### 3.8 Silencing of E2F1 in microglia improves neurological behaviour function in mice following CIRI injury

To further confirm the impact of E2F1 in microglia on neurological behaviour function in mice following CIRI injury, an adenovirus carrying E2F1 shRNA was constructed. The expression of Cre recombinase in the adenovirus was under the control of the microglia‐specific Cx3cr1 promoter, indicating that the E2F1 shRNA carried would only be activated in cells expressing Cx3cr1 (i.e., microglia). Through stereotactic injection, the AVV was delivered into the cerebral cortex of Cx3cr1‐Cre male mice, followed by observing the effects of silencing E2F1 on neurological behaviour function after constructing the CIRI mouse model.

**FIGURE 9:**
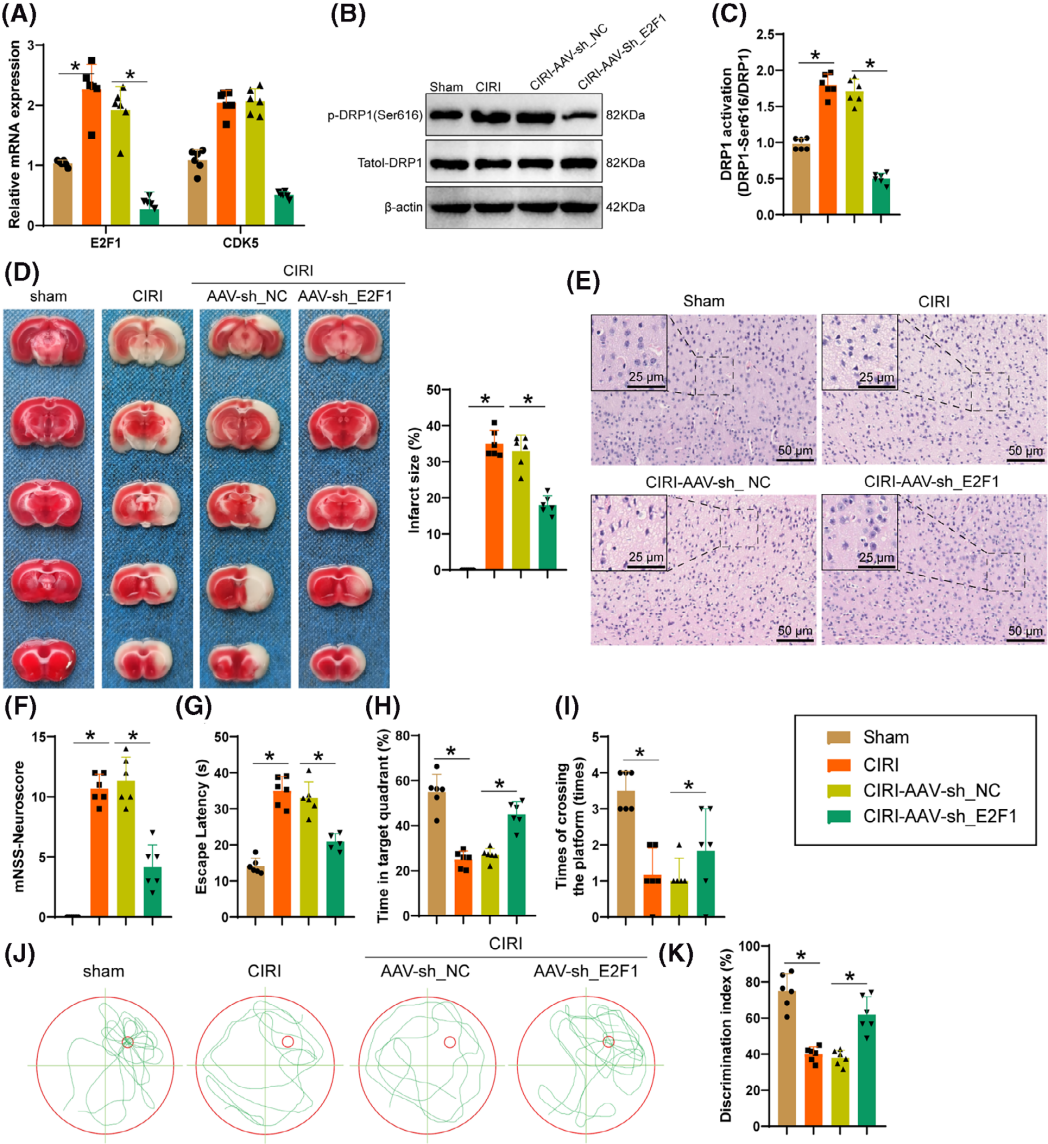
Impact of E2F1 silencing in microglia on neurobehavioral functions in mice following CIRI injury. (A) RT‐qPCR analysis of E2F1 and CDK5 expression levels in brain tissue. (B, C) Western Blot analysis of DRP1 activation in brain tissue. (D) Quantitative assessment of TCC staining and infarct area in the mouse brain. (E) H&E staining of the cerebral cortex in mice. Scale bar = 50/25 µm. (F) Evaluation of neurological deficits in mice through the mNSS analysis. (G) Delay in reaching the hidden platform on the 6th day for mice. (H) Time spent in the target quadrant during the probe trial on the 7th day in seconds. (I) Number of crossings over the target platform location by mice on the 7th day during the probe trial. (J) Representative swim paths of mice on the 7th day. (K) Percentage of time spent exploring the novel object in the novel object recognition test phase. Each group comprises six mice, *p < .05.

Evidenced by RT‐qPCR and Western blot data (Figure 9A–C), it was found that following E2F1 silencing, the levels of E2F1 and CDK5 in brain tissue significantly decreased, along with a notable reduction in the phosphorylation level of DRP1 at the Ser616 site. TEM images indicated that after silencing E2F1, fragmentation and vacuolization of mitochondria in the mouse cerebral cortex markedly reduced (Figure S6A,B), while lysosomes increased significantly in brain tissue (Figure S6C,D). TUNEL staining experiments exhibited a substantial decline in neuronal apoptosis rate in mouse brain tissue after silencing E2F1 (Figure S6E,F), suggesting that silencing E2F1 in microglia mitigates mitochondrial dysfunction in mice and reduces neuronal damage.

The outcomes of TTC and H&E staining (Figure 9D,E) displayed that silencing E2F1 reduced the infarct area and inflammatory infiltration in mouse brain tissue. Additionally, E2F1 silencing alleviated neuronal functional loss (Figure 9F). Assessment through the MWM test, a commonly utilized technique for assessing spatial memory, illustrated that mice with silenced E2F1 reduced navigation time towards the hidden platform (Figure 9G), prolonged their stay within the specific quadrant (Figure 9H), and completed more platform crossings on the 7th day of spatial probe test (Figure 9I). Representative swimming trajectories from the probe test are shown in Figure 9J. Following this, by examining the memory capacity of mice through novel object recognition testing, it was observed (Figure 9K) that mice treated with CIRI illustrated a reduction in the duration allocated to investigating the novel object, whereas mice with silenced E2F1 significantly preferred the novel object, indicating an enhancement in the mice's ability to recognize and remember. In summary, the silencing of E2F1 in astrocytes can improve the neurobehavioral function of mice following CIRI damage.

## 4 DISCUSSION

CIRI is a severe neurological condition involving various cellular and molecular changes.^58^, ^59^, ^60^ Mitochondria, crucial energy production centres and cellular regulators hold a crucial position in the occurrence and progression of CIRI.^5^, ^13^, ^61^ Previous studies have demonstrated that CIRI leads to mitochondrial dysfunction, manifested by reduced ATP synthesis, increased ROS production, and mitochondrial membrane potential loss, ultimately resulting in cell death and tissue damage.^7^, ^14^, ^62^ This study further validates the crucial role of mitochondrial dysfunction in CIRI and systematically investigates its regulatory mechanisms, providing new insights into therapeutic strategies.

Recent research indicates that the transcription factors E2F1 and CDK5 exert important influences in the nervous system, specifically in the onset and progression of CIRI.^29^ E2F1, as a key cell cycle regulator, has been shown to play essential roles in a range of neurological disorders like stroke, Parkinson's, and Alzheimer's, controlling multiple signalling pathways, including cell apoptosis and inflammatory responses.^63^ Therefore, E2F1 holds significant value in drug development. However, due to the broad regulatory pathways of E2F1, therapeutic approaches based on these studies primarily involve small molecule inhibition or gene silencing of downstream genes of E2F1.^64^ CDK5, a neuron‐specific kinase, plays essential roles in neuronal development and synaptic plasticity; however, its precise mechanisms of action in CIRI remain unclear.^34^, ^35^, ^65^ This study, using single‐cell transcriptome sequencing, provides the first evidence of the specific expression changes of E2F1 and CDK5 in CIRI, laying the foundation for further investigation into their mechanisms.

This study demonstrates that E2F1 promotes CDK5‐mediated phosphorylation of DRP1, which induces mitochondrial fission and inhibits mitophagy. This mechanism leads to mitochondrial structural damage and excessive ROS accumulation in microglial cells, further triggering apoptosis and inflammation. Previous studies have highlighted the critical role of DRP1 in ischemic brain injury.^66^ Specifically, inhibiting DRP1 activity significantly reduces infarct size and improves neurological function after ischemic injury.^67^ Furthermore, overactivation of DRP1 has been implicated as a central regulatory factor in mitochondrial dysfunction in various neurodegenerative diseases and ischemic brain injury,^68^ aligning with the findings of this study. Additionally, it has been suggested that upstream regulators such as CDK5 and GSK3β are involved in the activation of DRP1, which mediates mitochondrial fragmentation and exacerbates ischemic damage.^66^ This study further enriches the understanding of DRP1's role in ischemic injury, identifying E2F1/CDK5 as critical upstream regulators that modulate DRP1 activation.

Mitochondrial dysfunction and ROS accumulation are recognized as major contributors to the inflammatory response in CIRI.^69^ Excessive mitochondrial fission and ROS generation, particularly induced by activated DRP1, have been shown to play a critical role in this process after CIRI and ODG/R.^13^ ROS not only directly damage cellular structures but also activate key signalling pathways, including NF‐κB, which subsequently induces the release of proinflammatory cytokines such as TNF‐α, IL‐6, and IL‐1β, aggravating neuronal damage.^70^ These findings are consistent with our results, which also highlight the significant impact of DRP1‐induced mitochondrial fragmentation and ROS accumulation on microglial inflammation during CIRI. Moreover, studies have suggested that maintaining mitochondrial mass balance and reducing ROS accumulation by either limiting ROS production or enhancing ROS clearance may improve the prognosis of CIRI.^13^ Our study further supports this notion by demonstrating that inhibiting DRP1 activity could potentially reduce ROS generation, thus alleviating inflammation and mitigating neuronal damage. Additionally, other research has shown that mitochondrial calpain‐1 contributes to ROS production, triggering inflammatory responses and myocardial dysfunction during endotoxemia,^71^ which may be similarly relevant in the context of CIRI. Together, these findings underline the crucial role of mitochondrial dysfunction and ROS accumulation in the pathogenesis of CIRI and suggest potential therapeutic strategies targeting these pathways to reduce inflammation and improve outcomes.

ROS have been suggested as key mediators of microglial activation, although the precise mechanisms remain not fully understood.^72^ Through microglia‐neuron co‐culture experiments, this study demonstrates that the E2F1/CDK5 axis induces mitochondrial fission and ROS accumulation, leading to significant neuronal toxicity, including reduced survival rates and increased apoptosis. Silencing E2F1 effectively alleviated neuronal toxicity, while overexpression of CDK5 reversed these protective effects. Previous studies have implicated ROS and inflammatory cytokines as critical mechanisms underlying neuronal toxicity during microglial activation.^73^, ^74^, ^75^ This study further elucidates the regulatory role of the E2F1/CDK5/DRP1 axis in this process, highlighting its contribution to mitochondrial dysfunction and oxidative stress, and enriching the theoretical understanding of the mechanisms of neuronal toxicity in neuroinflammation.

In a CIRI mouse model, silencing the E2F1 gene significantly improved neurological function, including enhanced spatial memory and learning abilities, reduced infarct size, and decreased neuronal apoptosis rates. These findings are consistent with the protective effects of DRP1 inhibitors or ROS scavengers reported in other studies.^76^, ^77^, ^78^ This study further validates the therapeutic potential of the E2F1/CDK5/DRP1 axis and supports the development of precise intervention strategies targeting this pathway. It also provides a solid foundation for exploring the clinical translation of these findings.

Although this study elucidates the critical role of the E2F1/CDK5/DRP1 axis in CIRI, several limitations remain. First, the study primarily relies on mouse models, and future studies should incorporate more advanced experimental systems, such as humanized models or organoids, to validate the applicability of these mechanisms in humans. Additionally, variations in E2F1 and CDK5 expression levels between individuals may influence therapeutic outcomes, necessitating further investigations using multi‐omics approaches.

From a scientific perspective, this study provides new insights into the pathogenesis of CIRI, particularly in the regulation of mitochondrial fission and mitophagy, offering valuable clues for subsequent research. From a clinical perspective, the study highlights the E2F1/CDK5/DRP1 pathway as a promising target for drug development, with the potential to improve patient outcomes by mitigating mitochondrial dysfunction and inflammatory responses. Future research should also explore how these regulatory mechanisms can be integrated into precision medicine to develop more effective individualized treatments for CIRI patients.

## 5 CONCLUSION

**FIGURE 10:**
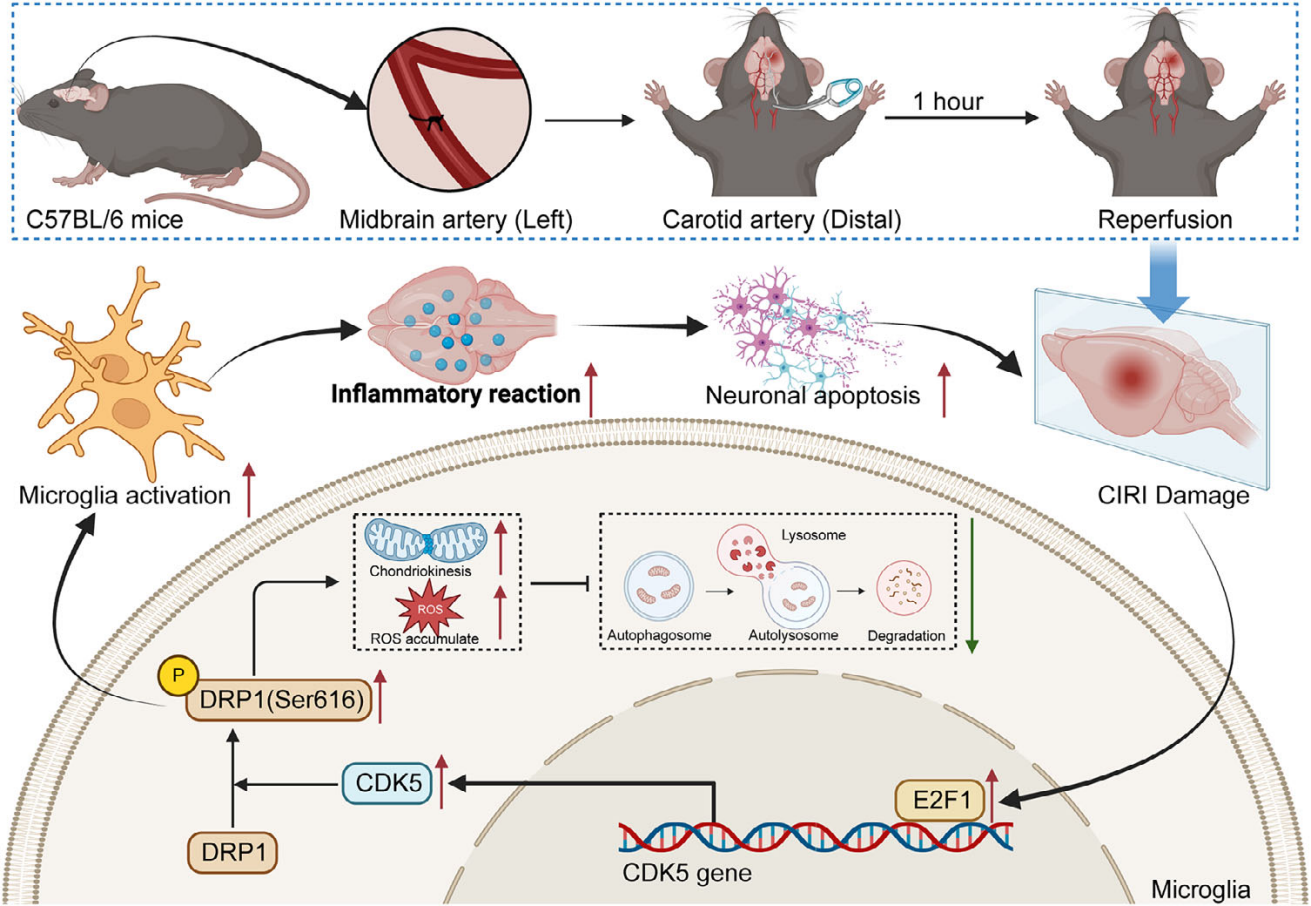
Schematic representation of the mechanism by E2F1/CDK5/DRP1 mediating microglial mitochondrial division and autophagy impact on CIRI.

According to the comprehensive research results, we can initially infer the subsequent deductions (Figure 10): in microglia, E2F1 activates CDK5 transcriptionally to promote DRP1 phosphorylation, thereby enhancing mitochondrial division while inhibiting mitophagy, leading to impaired mitochondrial function. This process triggers microglia activation, initiating brain tissue inflammation, accelerating neuronal apoptosis, and ultimately exacerbating the damage caused by CIRI. This research provides important perspectives on the molecular mechanisms underlying CIRI, particularly revealing the key role of E2F1 in mediating microglial mitochondrial division and autophagy through regulating CDK5‐DRP1 phosphorylation. This discovery not only advances our insight into the pathogenic pathways of CIRI but also lays the groundwork for pinpointing fresh treatment targets.

Although mouse and cell culture models were utilized, these models may not fully recapitulate the complexity of human CIRI. The biological background of human disease may vary due to genetic and environmental factors; therefore, research findings need further validation in human or more physiologically relevant models. Treatment strategies proposed in the study should consider the specificity of target molecules and potential side effects. Building on these findings, prospective clinical experiments may be organized to validate the efficiency and safety of targeting the E2F1‐CDK5‐DRP1 signalling axis in treating human CIRI.

## AUTHOR CONTRIBUTIONS

Ya‐jing Yuan, Tingting Chen, and Yan‐ling Yang contributed equally as co‐first authors to this study. Ya‐jing Yuan and Tingting Chen designed and performed the experiments, analyzed data, and drafted the manuscript. Yan‐ling Yang conducted the bioinformatics analysis and contributed to the manuscript revision. Hao‐nan Han supported the experimental design and provided technical guidance. Li‐ming Xu conceptualized the study, supervised the project, and critically revised the manuscript for intellectual content. All authors reviewed and approved the final manuscript.

## CONFLICT OF INTEREST STATEMENT

The author declares no conflict of interest.

## ETHICS STATEMENT

All animal experiments were approved by the The Animal Ethical and Welfare Committee of Tianjin Medical University Cancer Institute and Hospital.

## Supporting information



Supporting Information

Supporting Information

Supporting Information

Supporting Information

Supporting Information

Supporting Information

Supporting Information

## Data Availability

All data are available from the corresponding author upon reasonable request.
