# Supplementary material for: E2F1/CDK5/DRP1 axis mediates microglial mitochondrial division and autophagy in the pathogenesis of cerebral ischemia‐reperfusion injury
Source: Clin Transl Med. 2025 Feb 19;15(2):e70197. doi: 10.1002/ctm2.70197 (PMC11836619; doi:10.1002/ctm2.70197)
Supplement: Supplementary file 1 — Supporting Information [file CTM2-15-e70197-s005.docx]

**Table S1. RT-qPCR primer sequences.**

| **Gene** | **Primer Sequence(5'-3')** |
| --- | --- |
| E2F1 | Forward:5'-GCATTGACTGACTGCCTTGC-3'  Reverse:5'-AAAGCCTGAGCGTGCACTAA-3' |
| CDK5 | Forward:5'-AAGCCCTACCCAATGTACCC-3'  Reverse:5'-GCCTATGGGGGACAGAAGTC-3' |
| GAPDH | Forward:5'-CCCTTAAGAGGGATGCTGCC-3'  Reverse:5'-TACGGCCAAATCCGTTCACA-3' |

**Table S2. Differential expression of genes in Astrocytes and Neurons**

| **Gene** | **Astrocytes** | | **Neurons** | |
| --- | --- | --- | --- | --- |
|  | Log2FC | p-Value | Log2FC | p-Value |
| E2f1 | 0.28 | 0.06 | 0.49 | 0.71 |
| Cdk5 | 0.27 | 0.10 | 0.34 | 0.12 |
