# Supplementary figures and images for: E2F1/CDK5/DRP1 axis mediates microglial mitochondrial division and autophagy in the pathogenesis of cerebral ischemia‐reperfusion injury
Source: Clin Transl Med. 2025 Feb 19;15(2):e70197. doi: 10.1002/ctm2.70197 (PMC11836619; doi:10.1002/ctm2.70197)

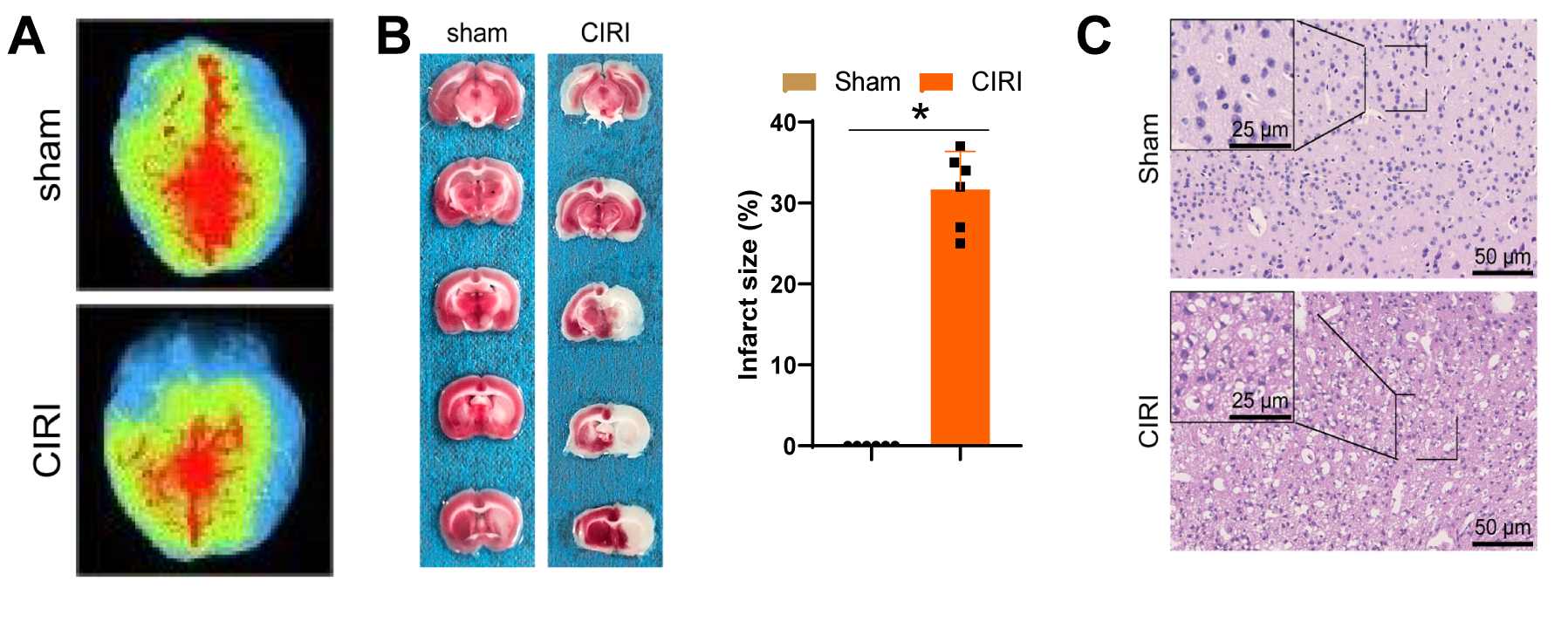

Supplement: Supplementary file 2 — Supporting Information [file CTM2-15-e70197-s007.tif]

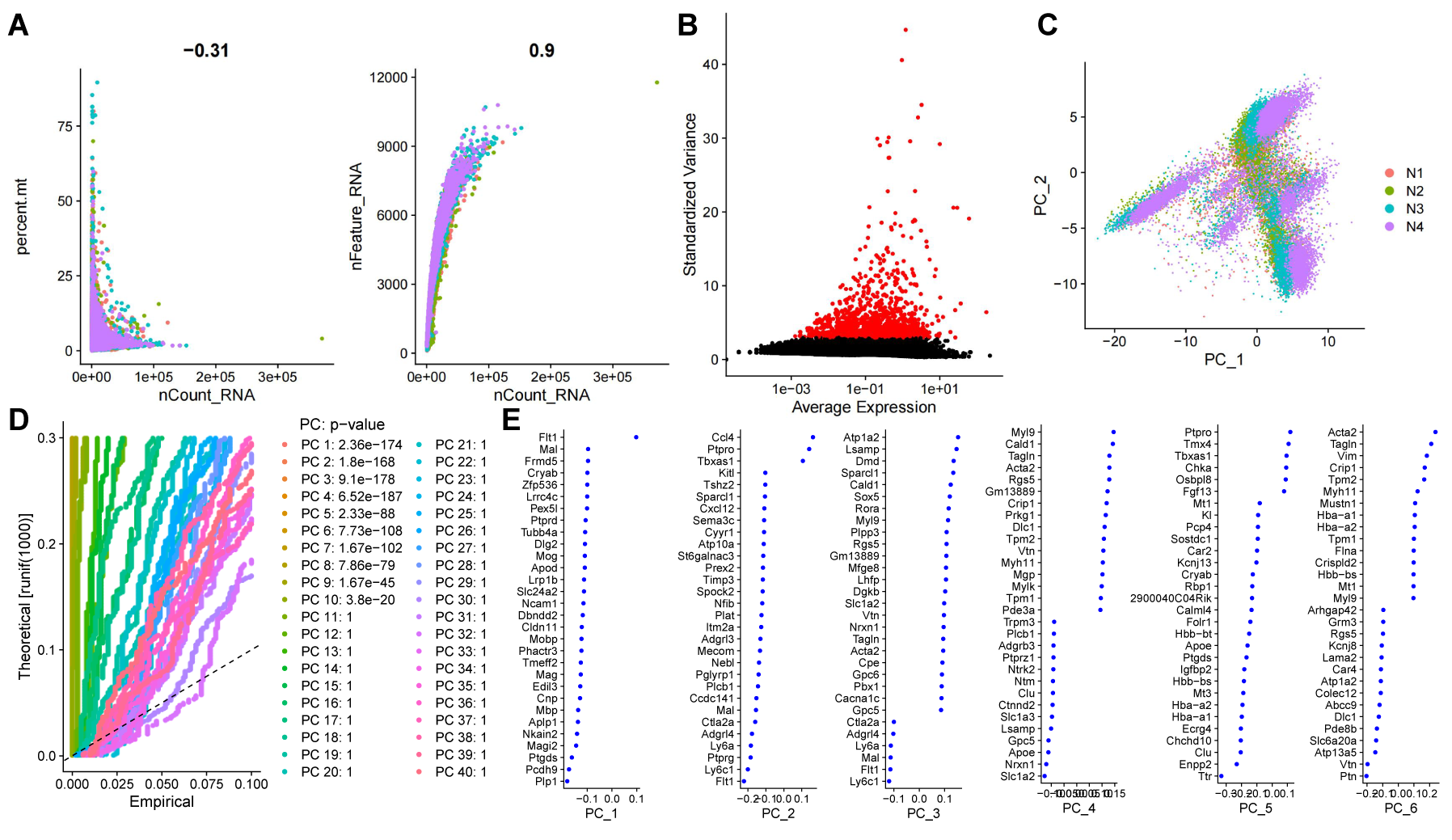

Supplement: Supplementary file 3 — Supporting Information [file CTM2-15-e70197-s002.tif]

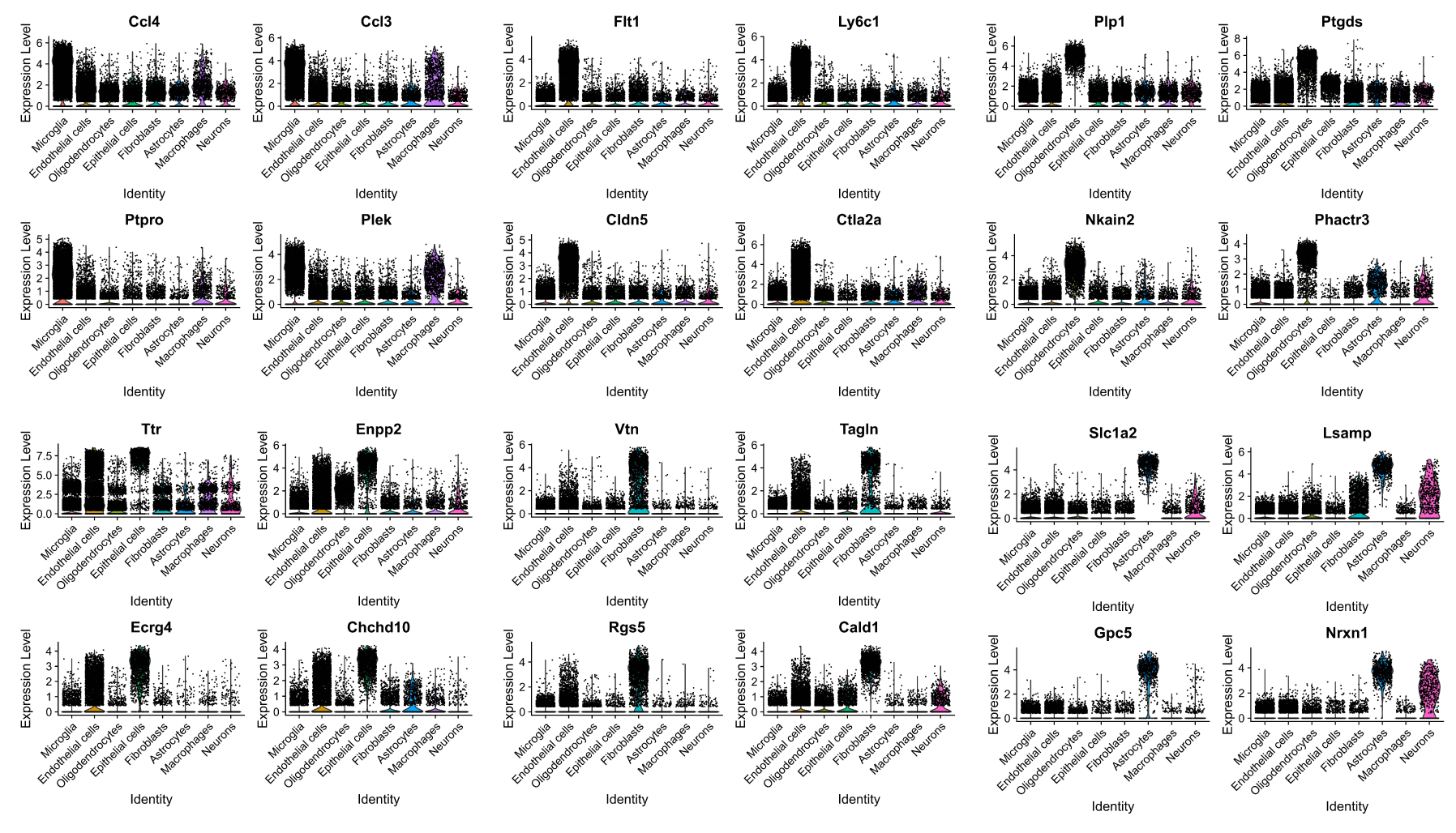

Supplement: Supplementary file 4 — Supporting Information [file CTM2-15-e70197-s004.tif]

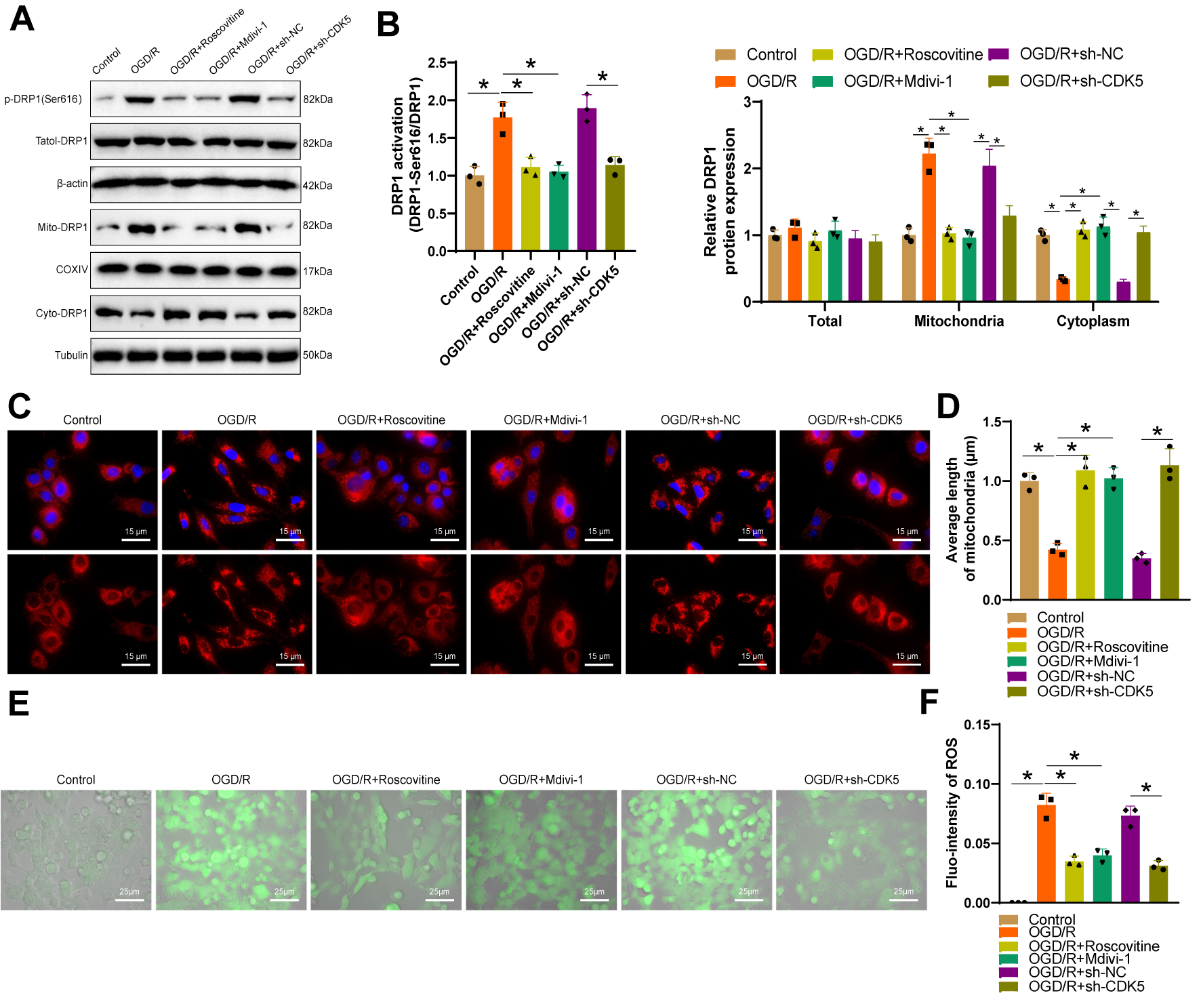

Supplement: Supplementary file 5 — Supporting Information [file CTM2-15-e70197-s003.tif]

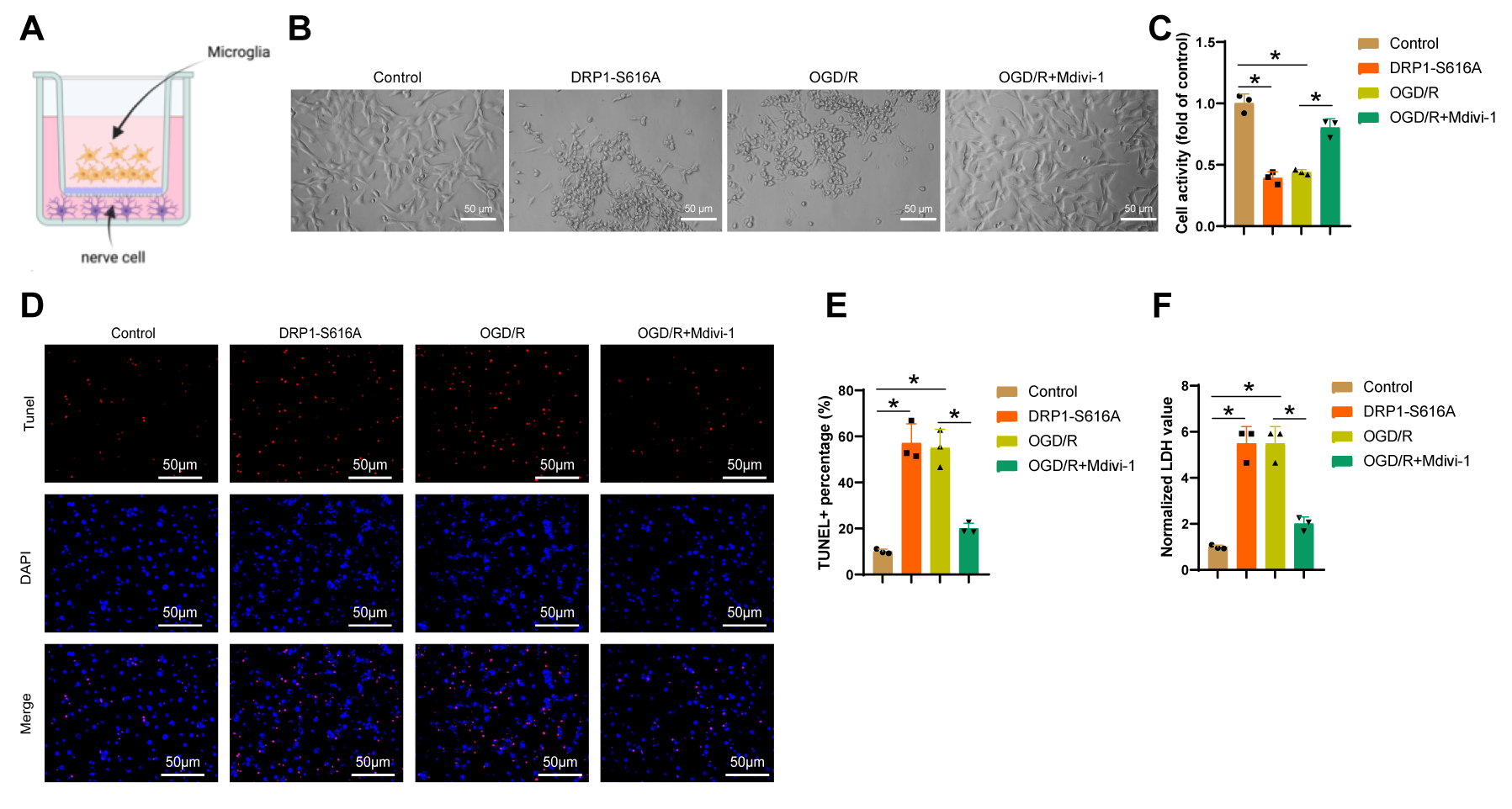

Supplement: Supplementary file 6 — Supporting Information [file CTM2-15-e70197-s001.tif]

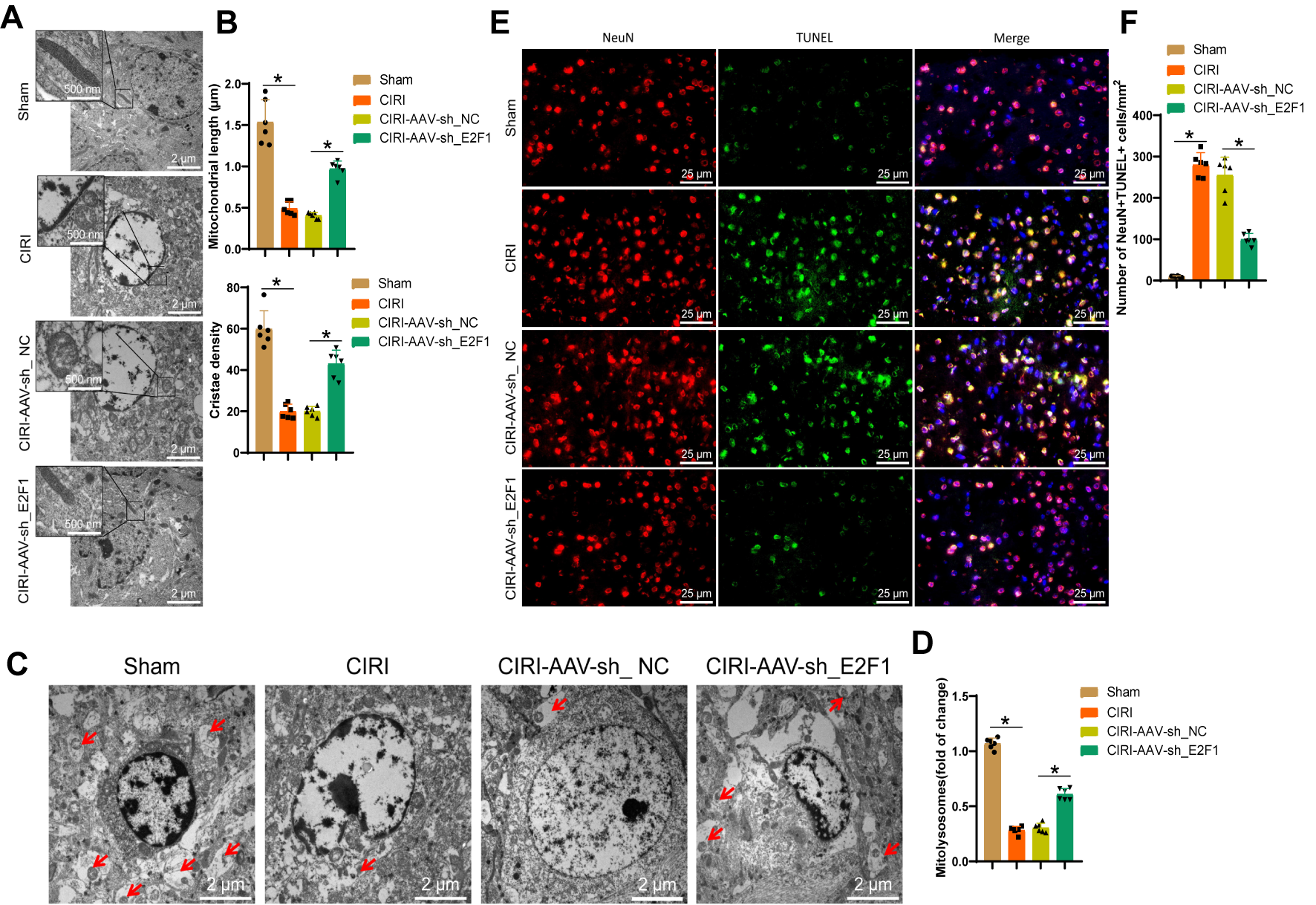

Supplement: Supplementary file 7 — Supporting Information [file CTM2-15-e70197-s006.tif]
